# A Review of Predictive Analytics Models in the Oil and Gas Industries

**DOI:** 10.3390/s24124013

**Published:** 2024-06-20

**Authors:** Putri Azmira R Azmi, Marina Yusoff, Mohamad Taufik Mohd Sallehud-din

**Affiliations:** 1 College of Computing, Informatics and Mathematics, Universiti Teknologi MARA (UiTM), Shah Alam 40450, Selangor, Malaysia; 2 Institute for Big Data Analytics and Artificial Intelligence (IBDAAI), Universiti Teknologi MARA (UiTM), Shah Alam 40450, Selangor, Malaysia; 3 Faculty of Business, Sohar University, Sohar 311, Oman; 4 PETRONAS Research Sdn Bhd, Petronas Research & Scientitic, Jln Ayer Hitam, Bangi Government and Private Training Centre Area, Bandar Baru Bangi 43000, Selangor, Malaysia; mohdtaufik@petronas.com

**Keywords:** classification, clustering, machine learning, oil and gas, predictive analytics

## Abstract

Enhancing the management and monitoring of oil and gas processes demands the development of precise predictive analytic techniques. Over the past two years, oil and its prediction have advanced significantly using conventional and modern machine learning techniques. Several review articles detail the developments in predictive maintenance and the technical and non-technical aspects of influencing the uptake of big data. The absence of references for machine learning techniques impacts the effective optimization of predictive analytics in the oil and gas sectors. This review paper offers readers thorough information on the latest machine learning methods utilized in this industry’s predictive analytical modeling. This review covers different forms of machine learning techniques used in predictive analytical modeling from 2021 to 2023 (91 articles). It provides an overview of the details of the papers that were reviewed, describing the model’s categories, the data’s temporality, field, and name, the dataset’s type, predictive analytics (classification, clustering, or prediction), the models’ input and output parameters, the performance metrics, the optimal model, and the model’s benefits and drawbacks. In addition, suggestions for future research directions to provide insights into the potential applications of the associated knowledge. This review can serve as a guide to enhance the effectiveness of predictive analytics models in the oil and gas industries.

## 1. Introduction

As stated in the International Energy Agency’s 2020 report, the oil and gas (O&G) sector plays an important role in the global economy and substantially contributes to fulfilling the world’s energy needs. The efficient management and optimization of operations within this sector are important for ensuring a dependable energy supply, mitigating environmental impacts, and maximizing economic returns [1,2]. Predictive analytics uses statistical modeling, data mining, and ML to predict outcomes based on past data [3,4]. This approach has gained popularity and facilitates decision-making by considering qualitative and quantitative data. The practice involves evaluating several factors to determine the relevance of predictions, as highlighted by Sharma and Villányi [5]. Various well-known predictive analytics models, such as classification, clustering [6], and prediction models, are utilized in this context [7]. Predictive analytics is crucial in real-world scenarios within the O&G industry. Examples include its application in optimizing drilling operations, which is employed to adapt to the detection and identification of drill pipe stuck-up events [8]. In pipeline risk assessment, predictive analytics also validates the effectiveness of algorithms for calculating the need for strain in a pipe [9]. Furthermore, predictive analytics is employed in exploration and production to detect and classify events to minimize downtime, reduce maintenance costs, and prevent damage to installations in oil wells [10].

Predictive analytics in the O&G field can be better understood by in-depth knowledge of its past, present, and future situations. This includes pipelines, wells, and gas and oil models. Several articles describe the advancements in predictive maintenance and the technical and non-technical factors affecting significant data implementation. The review article recommended further research on integrating AI with other state-of-the-art technologies. AI has the potential to revolutionize maintenance techniques, and its ongoing development will indeed influence how the O&G sector develops in the future [11]. This is because there are still issues with AI methods and tools, such as overfitting, coincidence effects, and overtraining [12].

**Figure 1:**
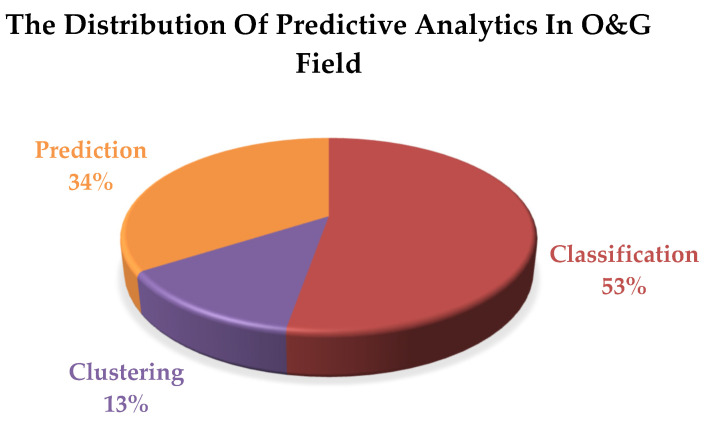
Distribution of the predictive analytics model in the O&G field.

Furthermore, many studies have been conducted using various simulation methodologies for quantitative and qualitative predictive analytics in the O&G field in terms of classification, clustering, and prediction. In the last two years, ML models have been extensively applied to O&G predictive analytics to address the shortcomings of traditional numerical models. Figure 1 presents a pie chart of the distribution of the predictive analytics model.

Figure 1 illustrates the three categories of predictive analytics applied in the study using ML and AI techniques. A little over 13% of clustering studies have employed modeling methods. Many of these do not require clustering studies because there is enough supervised labeling data, which leads to 53% of researchers favoring classification.

**Figure 2:**
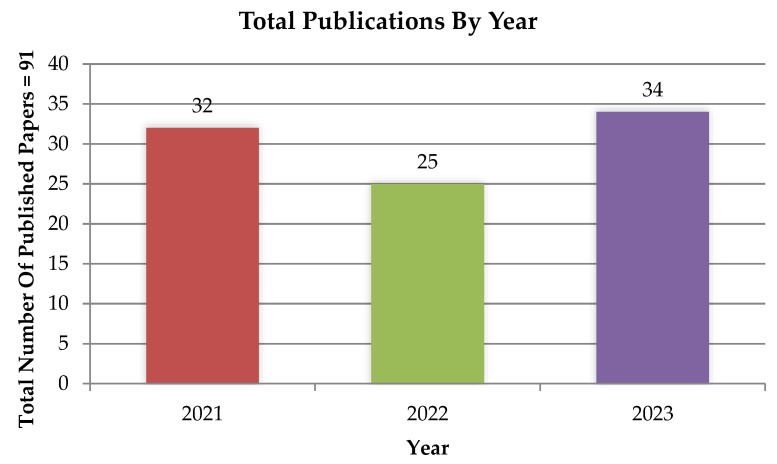
Total of predictive analytics models in the O&G field by year.

Recently, modern artificial intelligence models, such as ANN, Deep Learning (DL), Fuzzy Logic, Decision Tree (DT), RF, and hybrid models have been implemented to model the O&G domain, such as a review of 91 publications and a bibliography on the use of AI in the O&G field. Figure 2 shows that, in recent decades, this field of research has increased. Nevertheless, additional studies on predictive analytics models and datasets are required to identify the suitability of the model and dataset for incorporating diverse mathematical and statistical elements alongside heuristic and arithmetic methods. The use of AI has been widely utilized in various fields, such as science [13,14,15], energy [16,17,18], and economics [19,20,21]. Some examples include ML techniques [22,23,24], ensemble techniques [25,26], soft computing techniques [27,28], statistical techniques [29], and fuzzy-based systems [30]. The effective application of AI in several O&G domains, such as gas [31], pipeline [32], crude oil [33], oxyhydrogen gas retrofit [34], and transformer oil [35], has received increased interest in the last few years.

Predicting the performance and production of O&G has consistently presented a challenge. The imperative to create resilient prediction methods is driven by the desire for enhanced financial viability and superior technical outcomes [36]. As a critical sector, the O&G industry faces complex challenges, ranging from volatile market conditions to operational uncertainties and safety concerns. Its transformative potential is to revolutionize operations, enhance efficiency, and mitigate risks. 

Predictive analytics offers a powerful toolset to address these challenges and unlock numerous benefits. For instance, proactive decision-making by O&G engineers is made possible by operational efficiency from real-time data analysis. This helps organizations spot problems before they escalate, optimize resource utilization, and streamline processes. In addition, cost reduction can help O&G companies be cost-effective by optimizing resource allocation, reducing waste, and enhancing overall resource efficiency through insights from predictive analytics. Numerous studies have explored and documented AI’s effectiveness in modeling O&G over the last three years. Many initial efforts comprised basic and conventional AI techniques, including perceptron-based Artificial Neural Networks (ANNs) [37,38,39].

The subsequent sections provide thorough descriptions and in-depth analyses of the utilization of ML models for O&G prediction. Given the detailed exploration in these sections, providing additional information on this topic in the form of a literature review would be redundant and unnecessary. While some comprehensive analyses of O&G modeling utilizing ML models have been conducted, like the most current research conducted by Taha and Mansour [40], it has been suggested that optimized machine learning techniques and data transformation methods can increase the precision of the faulty power transformer prediction for Dissolved Gas Analysis (DGA) in the O&G field. Additionally, the aim of this paper is to discuss the most recent advancements, progress, constraints, and difficulties related to complex AI techniques for O&G data management. Because of this, researchers, petroleum engineers, and environmentalists attracted by the possible uses of AI within the oil and gas industry represent the target audience for this article.

## 2. Predicted Analytics Models for O&G

### 2.1. Application of Artificial Neural Network Models

This model is a computational framework that imitates how data are processed and analyzed in the cognitive structure of humans [41]. Neural networks accumulate their understanding by identifying patterns and relationships in data through experiential learning [42]. The ANN’s architecture consists of three essential elements, including input, process, and output, and its functionality is predominantly determined by the interconnections between these elements and the role of connections in natural processing [43]. An ANN aims to convert inputs into meaningful outputs [44]. Before being transmitted to the output layer, data are initially introduced into the layer of input, which processes it before forwarding it to the hidden layer. Each layer is made up of neurons that resemble computational units. These neurons use activation functions like sigmoid, linear, tanh, and o analyze each data record. Several optimizers are available to improve neural network performance by iteratively adjusting network weights based on training data [44,45].

The research has extensively explored the versatile application of ANN models for predicting O&G properties across diverse domains. Qin et al. [46] thoroughly explored non-temporal data from a buried gas pipeline, employing various algorithms with a combination of ANN and metaheuristics models such as the Quantum Particle Swarm Optimization-Artificial Neural Network, Weighted Quantum Particle Swarm Optimization-Artificial Neural Network (QPSO-ANN), and Levy Flight Quantum Particle Swarm Optimization-Artificial Neural Network (LWQPSO-ANN). The study focused on predicting crater width, with important parameters for the prediction of buried pipelines, such as pipe diameter (mm), operating pressure (MPa), cover depth (m), and crater width (m). In this work, LWQPSO-ANN outperformed other methods by more than 95%. 

Meanwhile, in another study on non-temporal pipeline conditions, a range of ML algorithms, including ANN, Support Vector Machine (SVM), Ensemble Learning (EL), and Support Vector Regression (SVR), were used [47]. Their investigation included elements impacting corrosion defect depth, such as CO_2_ levels, temperature, pH, liquid velocity, pressure, stress, glycol concentration, H_2_S levels, organic acid content, oil type, water chemistry, and hydraulic diameter. The emphasis on the ANN was evident, indicating that it is a skilled navigator of the complex network of variables affecting pipeline corrosion. In the complicated landscape of well-data analysis, Sami and Ibrahim [48] utilized non-temporal datasets from Middle East fields, concentrating on vertical wells. Random Forest (RF), k-nearest Neighbors (KNN), and ANNs were used to predict the bottom-hole pressure flowing (Pwf) through vertical petroleum wells. The preference for the ANN spotlighted its efficacy in modeling intricate relationships within well data, as underscored by evaluation metrics such as the Mean Squared Error (MSE) and Coefficient of Determination (R^2^) The proposed method that used R^2^ values for training and testing were 97% and 93% respectively, significantly higher than the models implemented in the study.

Moreover, Qayyum Chohan et al. [49] constructed non-temporal datasets using ML algorithms like the ANN, Least Square Boosting (LSB), and Bagging for the prediction of oil using 2600 samples from oil shales. The input parameters that were used in the study are air molar flowrate, illite silica, carbon, hydrogen content, feed preheater temp, and air preheater temp. Through a coefficient of correlation of 99.6% for oil yield and 99.9% for carbon dioxide, the Root Mean Squared Error (RMSE) evaluation metric was highlighted, emphasizing the applicability of ANNs in interpreting the complex factors influencing oil yield and carbon dioxide emissions in complex processes. The suggested model outperformed other models in terms of accuracy. A set of ML methods, including NB+KNN, DT, RF, SVM, and ANN, were applied to 769 temporal data samples related to ocean slick signs in the surrounding area of the exploration site [50]. The study’s emphasis on ANNs amidst this array of algorithms underscored its pivotal role in discerning Sea-Surface Petroleum Signatures. Although the specific parameters of the ocean slick signature were not explicitly stated, the study spotlighted the ANN’s prowess in unraveling patterns related to oil detection in dynamic ocean conditions with an accuracy of 90%. However, the proposed model did not give significant results for classifying ocean slick signatures.

Several machine learning models were used in the study, including Partial Least Squares (PLS), Deep Neural Network (DNN), Feature Projection Model (FPM), Feature Projection-Deep Neural Network (FP-DNN), and Feature Projection-PLS (FP-PLS) [51]. The study looked at long-distance pipelines without considering time. The dataset consisted of 2093 samples, and the prediction task included characteristics such as the original total oil length, inner dimensions, pipeline length, Reynolds quantity, comparable length, and actual combined oil length. The assessment parameter employed was RMSE, and the DNN model displayed an RMSE of 146%. The research showed that the error rate was the highest and least convincing one, indicating that the model’s prediction accuracy must be increased. Utilizing the ASPEN HYSYS V11 process simulator, Mendoza et al. [52] used non-temporal analysis in crude oil processes. The study used the ANN and Genetic Algorithm (GA) to predict critical variables such as feed flow rate, gas product pressure, interstage gas discharge pressure, and centrifugal compressor isentropic efficiency, aiming to increase oil production. The ANN+GA model improved the performance of the predicted variable.

Shifting the focus to gas-phase pollutants, Sakhaei et al. [53] performed non-temporal research using proprietary data. The study used ANNs to estimate methanol, α-pinene, and hydrogen sulfide concentrations for gas-phase contamination removal in OLP-BTF and TLP-BTF. The ANN+PSO model, which used 104 samples, achieved a desired performance measurement using R^2^ of more than 99% indicating its effectiveness. The authors were prompted to contemplate possible improvements for practical implementations when the suggested model showed encouraging outcomes. ANN, Least Square Support Vector Machine (LSSVM), and Multi-Gene Genetic Programming (MGGP) were utilized in reservoir engineering to analyze temporal data for gas-aided gravity drainage (GAGD) [54]. Compared to the suggested strategy, with various input parameters and 223 samples, the ANN’s model showed 976% of R^2^ and 0.0520 of RMSE. In contrast, MGGP returned 89% (R^2^) and 0.0846 (RMSE). The study demonstrated the superiority of the ANN technique in reservoir prediction tasks.

Mao et al. (2022) investigated DGA datasets by combining Multivariate Time Series clustering approaches and graph neural networks (GNNs), moving on to transformer fault diagnosis in the temporal domain. The study concentrated on clustering H_2_, CH_4_, C_2_H_6_, C_2_H_4_, C_2_H_2_, CO, and CO_2_ using 1408 samples to diagnose power transformer defects. The MTGNN model attained an impressive 92% accuracy, demonstrating its efficacy in the spatiotemporal area of power transformer problem detection. In the context of non-temporal analysis within the field of crude oil, Wang et al. [33] studied contemporary research, employing an ANN and a hybrid Multilayer Perceptron with Backpropagation for prediction. The model used 172 samples and a variety of characteristics to estimate diffusion coefficients, including temperature, pressure, liquid viscosity, gas viscosity, liquid molar volume, gas molar volume, liquid molecular weight, gas molecular weight, and interfacial tension. Although the training and testing R^2^s were 88% and 89%, respectively, the proposed Multilayer Perceptron with Backpropagation model had less accuracy, and the hybrid technique did not deliver the expected improvement.

The study from Zhang et al. [55] experimented with the temporal crude oil and transportation system data using the GA with a backpropagation neural network for prediction. The model produced outstanding results with 509 samples, including numerous factors linked to the system’s temperature, pressure, and consumption, achieving 99% accuracy for energy and heat and 97% for power. The GA with a backpropagation neural network was highly influential in predicting the complicated dynamics of the crude oil system. In cooperation with the Egyptian General Petroleum Corporation (EGPC), Ismail et al. [56] conducted a temporal study of drilling activities. The model used Multilayer Perceptron (MLP) and the ANN for grouping and classification tasks based on epochs, age, formation, lithology, and fields for predicting gas routes and chimneys. Surprisingly, the MLP model achieved an RMSE of 0.10, indicating decreased error rates and surpassing other approaches for predicting drilling-related occurrences.

The Extreme Learning Machine (ELM), Elastic Net Linear, Linear Support Vector Regression (Linear-SVR), Multivariate Adaptive Regression Spline, Artificial Bee Colony, Particle Swarm Optimization (PSO), Differential Evolution, Simple Genetic Algorithm, Grey Wolf Optimizer (GWO), and Exponential Natural Evolution Strategies (xNES) are some of the models that Goliatt et al. [57] used in the temporal domain of shale gas exploration within the YuDong-Nan shale gas field. To estimate total organic carbon, the DE+ELM hybrid model produced an acceptable RMSE of 0.497 when predicting factors such as clay, K-feldspar, pyrite, and other elements. Nevertheless, GWO did not outperform the other approaches. In the temporal field of reservoir engineering, specifically within the North Sea’s “Gullfaks”. An MLP-LMA model was suggested by Amar et al. [58] to produce predictions for half-cycle time, shutdown, water alternating gas injection, and the amount of gas and water injected. The proposed approach outperformed the other two proxy models, achieving higher accuracy and much shorter simulation times. Table 1 lists research articles on predictive analytics in the O&G field using ANN models.

### 2.2. Application of Deep Learning Models

**Figure 3:**
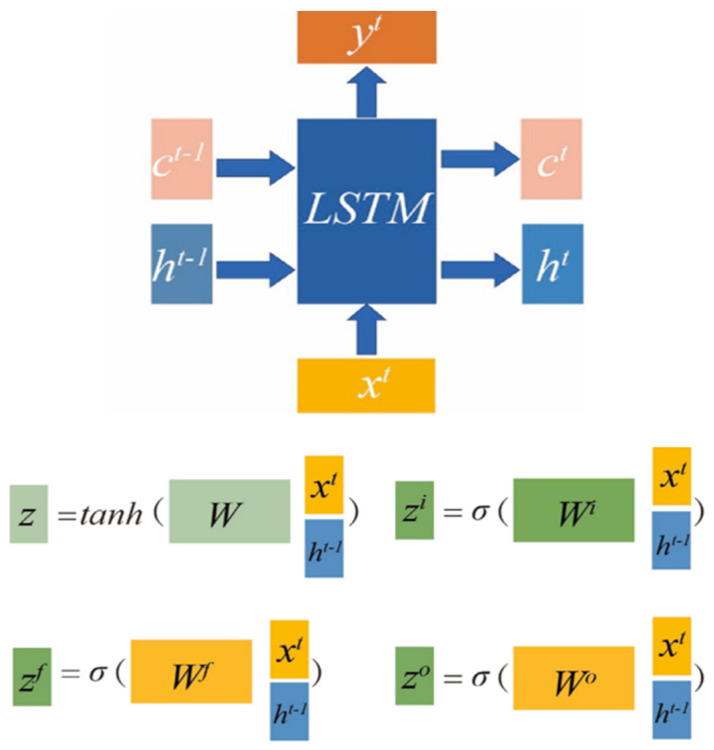
Internal Structure of LSTM [62].

The DL framework appears to beat several complex models based on DL and ML regarding the prediction accuracy [60]. It is more frequently utilized in algorithms for the life prediction of O&G equipment [61]. A layer of input, hidden layers, and an output layer contribute to a DL model. The parameters are assigned a value in the output layer using a neural network [43]. The most commonly used Deep Learning algorithms in gas pipeline research are the Conventional Neural Network (CNN) and LSTM [61]. Figure 3 shows the internal structure of LSTM model. The LSTM model’s ability to keep essential data for a longer period is one of its main benefits. Then, it can be applied to a wide range of tasks that require long-term memory. However, there are several constraints to consider while using the LSTM model. It’s important to realize that increasing the number of factors makes training more challenging [62].

Figure 3 shows the processes of the input series in both backward and forward directions. Bi-LSTM models can learn from the entire sequence context by collecting information about each sequence element from the past and future. They are highly suited for temporal data and producing precise predictions of ions in the sequence [62].

There are two transfer states in the LSTM model from Figure 3: a hidden state (*h^t^*) and a cell state (*c^t^*) [62]. The passed *c^t^* changes quite slowly. The output *c^t^* is passed from *c^t^*^−^^1^ in the previous state, with some added values [62]. However, there are typically significant variances in *h^t^* among nodes. The LSTM model used the current input of *x^t^* and *h^t^*^−^^1^ from the previous state to generate four states. Furthermore, *z^f^*, *z^i^*, and *z^o^* are accessible to a gating-control state with values between 0 and 1, derived by multiplying the splicing vector by the weight matrix and converting it by a sigmoid activation function. The tanh activation function converts z to a value between −1 and 1 [62]. 

This interest in Deep Learning is exemplified by a series of significant studies showcasing its applications. The success of MLSTM in this context was evident through robust evaluation metrics such as MAE and RMSE. Building on this, Werneck et al. [63] extended the 301 samples of temporal analysis to oil wells from the Metro Interstate Traffic Volume, Appliances Energy Prediction, and UNISIM-II-M-CO datasets, utilizing LSTM, Gated Recurrent Unit (GRU), and LSTM + Seq2Seq architectures for predicting oil production and pressure. The parameters used in the study to predict oil production and pressure are pressure (bottom-hole), water cut, gas–oil ratio, and gas–liquid ratio, which are considered in the ratios between fluid production (oil, gas, and water). Symmetric Mean Absolute Percentage Error (SMAPE), RMSE, and MAE are evaluation measures that demonstrate how well the models capture the dynamic characteristics of reservoirs. The LSTM + Seq2Seq and GRU2 architectures are the best models that the researchers have proposed because of the higher accuracy achieved. Nevertheless, the researchers recommend that future studies include another metaheuristic method, such as the GA.

In 2022, Wang et al. [61] shifted the focus to the Longmaxi Formation of the Sichuan Basin with 90,000 data samples for predicting the real-time pipeline crack. The study proposed the DCNN + LSTM, ANN, LSTM, Recurrent Neural Network (RNN), and SVR models for natural gas pipelines. The model showcases the impressive performance of the DCNN + LSTM with an accuracy of 99.37%, emphasizing the significance of LSTM in predicting shale gas production with robust evaluation metrics in the temporal well data setting. Antariksa et al. [64] used the West Natuna Basin dataset, which contains 11,497 samples, aligned with input parameters, such as deep and shallow resistivities (LLD and LLS), sonic (Vp), neutron-porosity (NPHI), density (RHOB), and gamma ray (GR), and one output parameter, well log data imputation, to apply LSTM and RF models to predict hydrocarbon production in the gas sector. This demonstrates that LSTM may be applied to the gas output forecast using metrics like R^2^, RMSE, and MSE. The suggested model provides 94% more accuracy. 

Another study explored the classification of non-temporal oil transformers using the DGA local power utilities and IEC TC10 datasets with 1530 samples. The research utilized KNN, SVM, and Extreme Gradient Boosting (XGBoost) to evaluate the model’s performance using measures including accuracy, precision, and recall. This shows the combination of the oversampling method, i.e., Synthetic Minority Oversampling Technique (SMOTE), and KNN (KNN+SMOTE) shows the performing accuracy of DGA and IEC TC10, which are 98% and 97%, respectively [65]. Barjouei et al. [66] studied non-temporal data from the Soroush and South Iran oil fields, analyzing 7245 samples and predicting factors such as choke size (D64), wellhead pressure (Pwh), oil specific gravity (γo), gas/liquid ratio, and wellhead choke. The study proposed a few models of DL, which are DL, DT, RF, ANNs, and SVR, revealing the superior performance of DL, has a greater accuracy R^2^ at 99% than the other models. Together, these studies highlight the adaptability of Deep Learning methods to handle temporal and non-temporal data in various O&G sector applications. The insights derived from these endeavors, specifically focusing on Deep Learning, contribute significantly to optimizing operations and decision-making processes in this critical industry.

The time domain of the reservoir focuses on the Volve and UNISIM-IIH oil fields and utilizes Long Short-Term Memory (LSTM) and GRU models for the classification of 3257 samples based on oil, gas, water, or pressure levels [67]. Regarding O&G forecasting, the GRU model emerged as the frontrunner. With an ideal R^2^ of 99%, the GRU model emerged as the leading model for O&G forecasting. This exceptional accuracy demonstrates the effectiveness of the suggested GRU model in predicting O&G activity within the given reservoir setting. In the analysis of non-temporal within the well domain, Wang et al. [68] applied various Faster R-CNN models, including Faster R-CNN_Res50, Faster R-CNN_Res50_DC, and Faster R-CNN_Res50_FPN, along with methods involving Edge detection and Cluster+Soft-NMS, utilizing Google Earth Imagery encompassing 439 samples. Their goal was to organize oil wells depending on breadth and height. The Faster R-CNN model with ClusterRPN obtained 71% precision. It is important to note that the suggested approach was less than 90% accurate and required more time to run than other models. Table 2 includes the published research on Deep Learning models for O&G predictive analytics.

### 2.3. Application of Fuzzy Logic and Neuro-Fuzzy Models

Neuro-fuzzy model is a hybrid model that leverages the respective advantages of both algorithms by combining two paradigms: Fuzzy Logic (FL) and ANNs [43]. Throughout several consecutive generations, FL’s function is to dynamically modify the crossover and mutation rates [69]. The ANN and FL were utilized to develop the renowned Adaptive Neuro-fuzzy Inference Systems (ANFIS) model [70]. In ANFIS, a neural network receives input from a fuzzy inference system. The ANFIS model is also computationally feasible, reducing the training time of the neural network [70].

The use of the ANFIS model to forecast the ruptured pressure of a faulty pipe utilizing the diameter of the pipeline, burst pressure, thickness of the pipe wall, defect depth, and defect width gave acceptable results, with corresponding RMSE, Mean Absolute Error (MAE), and R^2^ values of 98%, 69%, and 99%, respectively [71]. The ANFIS+Principal Component Analysis (PCA) is a proposed method that outdistanced other models and significantly improved the model’s accuracy. Another study on O&G predictive analytics focused on different research on O&G predictive analytics focused on the clustering that the ANN, SVR, and ANFIS suggested in their prediction extraction of oil from a heterogeneous reservoir using a 5-spot waterflood [44]. The study used 9000 non-temporal samples from the reservoir in Saudi Arabia, including the degree of reservoir heterogeneity (V), mobility ratio (M), permeability anisotropy ratio (kz/kx), wettability indicator (WI), production water cut (fw), and oil/water density ratio (DR) data to predict the waterflood’s mobile oil recovery efficiency (RFM). The ANN had better accuracy than the other models, with MAPE, MAE, MSE, and R^2^ values of 5.1666%, 0.0093, 0.0003, and 0.997, respectively, reducing the runtime by 0.8470 min. 

In contrast, only a small number of studies [72] studied the application of ANFIS in predictive analytics in the O&G sector. The discovered alternative ML models like ANFIS to model and use an ML approach to maximize the oil adoption capacity of functionalized magnetic nanoparticles. Other than ANFIS, the study also employed the Least Squares Support Vector Machine (LSSVM) with the hybridization of a metaheuristic model, which is the Cuckoo Search Algorithm (LSSVM-CSA), and Gene Expression Programming for non-temporal predictions using oil data. The study addressed parameters like mixing time (min), MNP dosage (g/L), and oil concentration (ppm) to predict oil adsorption capacity (mg/g adsorbent). A comparative performance investigation of the ANFIS, LSSVM-CSA, and Gene Expression Programming showed that the highest accuracy achieved was LSSVM-CSA. The proposed method performed better than the other two models, according to the R^2^, which was 99% for the best model. Another study revealed the viability of the Control Chart and RF for failure detection [73]. The temporal 50,000 samples from the 3W dataset were utilized. The parameters “normal”, “fault”, and “high fault” in this dataset were derived from the sensor’s real-time well and consisted of P-PDG, T-PDG, and T-PCK. Combining the Control Chart and RF method showed higher sensitivity (99%) and specificity (100%). The summary of previously published research on Fuzzy Logic and Neuro-fuzzy modeling in predictive analytics in the O&G field is shown in Table 3.

### 2.4. Application of Decision Tree, Random Forest, and Hybrid Models

Considerable attention has been given to integrating AI and a variety of ML models within the O&G sector, which has implications for reservoir engineering, pipeline integrity, drilling, and transformer defect prediction. DT can handle categorical and numerical information [79]. In several research publications, DT was used to develop models that predict output variable values based on multiple input variables, and this algorithm produced decisions depending on the training data it was trained on [80]. Regarding the area of pipeline failure risk prediction, Mazumder et al. [81] extended non-temporal applications by employing an array of models, including the KNN, DT, RF, Naïve Bayes (NB), AdaBoost, XGBoost, Light Gradient Boosting Machine (LGBM), and CatBoost. The study focused on crucial parameters like pipelines with failure risk, which are classified based on their diameter, wall thickness, defect depth, fault length, yield strength, final tensile strength, and operational pressure. Critical Resilient Interdependent Infrastructure Systems and Processes from the National Science Foundation have 959 data samples. The meticulous evaluation based on precision, recall, and mean accuracy identified XGBoost as the preferred model. The proposed model needs to improve its accuracy by 85%.

Liu et al. [82] researched a variety of models to address non-temporal pipeline failure defects with 1500 samples from well log data from North China, including the LR, Stochastic Gradient Descent, SVM, Gaussian Process Regression (GPR), Binary Search Tree Ensemble, Binary Decision Tree, Sine Window, and ANN. Their assessment criteria included MAE, MSE, and RMSE, with the ANN achieving an ideal R^2^ performance of 99% for training and 96% for testing, proving the efficiency of these models in resolving pipeline integrity problems based on accuracy. Shifting to reservoir engineering, Taha and Mansour [40] utilized 542 samples of temporal well log data from North China, featuring parameters like C_2_H_2_, C_2_H_6_, CH_4_, and H_2_. Their exploration incorporated ELM, SVM, KNN, DT, RF, and EL, specifically focusing on classifying the power transformer fault. Within this context, the EL with training and testing accuracy values were 78% and 84%, respectively. Thus, the performance accuracy was not above 90%. The researchers found that the best model’s results contributed significantly to the research. In the non-temporal domain, using the 3147 samples from DGA, Saroja et al. [83] applied an array of models for transformer fault classification, encompassing DT, Linear Discriminant Analysis (LDA), Gradient Boosting (GB), Ensemble Tree, LGBM, RF, KNN, NB, ANN, and LR. The accuracy of the aimed study was based on the gas parameters from the DGA dataset, which were C_2_H_2_, C_2_H_4_, C_2_H_6_, and CH_4_. Considering an accuracy rating of 99.29%, the Quadratic Discriminant Analysis (QDA) model was the performed model. In conclusion, for this research, the proposed model obtained the best precision for the classifier model. 

Extending the scope to gas type classification in transformer fault scenarios, Raj et al. [84] employed the DT model without a comparison to the alternative model. Their classification efforts centered around fault types using features like H_2_, CH_4_, C2H_6_, C_2_H_4_, and C_2_H_2_, with an accuracy of the DT of 62.9%, emerging as a model based on accuracy and Area Under Curve (AUC). For predicting faults in transformer oil, the current model exhibited potential, and the researcher recommended exploring opportunities for refinement to enhance overall efficacy. In drilling applications, Aslam et al. [85] navigated 1984 non-temporal samples from the 3W public database using several models, including LR, DT, RF, KNN, SMOTE, Explainable Artificial Intelligence (XAI), Shapley Additive Explanation (SHAP), and Local Interpretable Model-Agnostic Explanation (LIME). Relevant characteristics included P-PDG, P-TPT, T-TPT, P-MON-PCK, T-JUS, PCK, P-JUS-CKGL, T-JUS-CKGL, and QGL. Their thorough examination encompassed accuracy, recall, precision, F1 score, and AUC, eventually selecting RF as the best performance since the results for accuracy, recall, precision, F1 score, and AUC were, 1.00%, 99.6%, 99.64%, 99.91%, and 99.77%, respectively. The proposed model yielded remarkable results.

Turan and Jaschke [86] used a dataset of 2000 samples labeled with undesirable events, including P-PDG, P-TPT, T-TPT, P-MON-CKP, and T-JUS-CKP, to classify the 3W dataset using various algorithms such as LDA, QDA, Linear SVC, Logistic Regression (LR), Decision Tree (DT), RF, and Adaboost with a temporal perspective. The assessment measures used were F1 score and accuracy, with a particular emphasis on DT, which reached a significant accuracy of 97%. However, feature selection increased training time rather than improved accuracy. Remarkably, the proposed technique struggled to categorize class 2 due to limited data availability and label disputes based on estimated attributes. The other study focused on using the same dataset and utilized one-directional, CNN, RF, Graph Neural Network (GNN), and QDA models [87]. RF achieved a mean accuracy of 95%. The evaluation measures used were F1 score, accuracy, precision, and recall. Specifically, the study discovered that increasing the number of time frames enhanced mean accuracy. On the other hand, the temporal analysis of well data completed by Brønstad et al. [88] focused on 3W wells. The work employed ML models, namely RF and PCA. The combination of RF and PCA achieved an accuracy of 90%. The accuracy of the suggested strategy was over 95% in each of the distinct classes, indicating that it is a valuable way to identify several anomalous occurrences in well data.

Ben Jabeur et al. [89] used LGBM, CatBoost, XGBoost, RF, and a neural network to assess a dataset of 2687 samples connected to the temporal characteristics of WTI crude oil prices. The categorization challenge involved forecasting the movement of numerous financial indicators in connection to oil prices, including green energy resources, metals such as gold, silver, petroleum, soybeans, platinum, and copper, the Dollar Index, the Volatility Index, the Euro, the USD, and the Bitcoin. Accuracy and Area Under the Curve (AUC) were utilized as the assessment criteria. LGBM and RF fared better than the other algorithms in the research. The data imply that the suggested strategy is superior to established methods in forecasting complicated connections. Hassan Baabbad et al. [90] investigated the prediction of CO_2_ levels in shale gas reserves, emphasizing non-temporal factors. The study used ML algorithms like GB, RF, and Multiple Linear Regression (MLR) on a dataset of 1400 samples with a variety of features such as horizontal wellbore length, hydraulic fracture length, reservoir length, SRV fracture porosity, SRV fracture permeability, SRV fracture spacing, total production time, and fracture pressure. The performance was examined using MSE, and RF outperformed the other ML algorithms. The study emphasized the usefulness of RF as a superior approach in ML for forecasting CO_2_ levels in shale gas reserves compared to the other methods.

The study was evaluated by Alsaihati et al. using RF, ANNs, and Fuzzy Networks (FNs) on real-time well data with 8983 samples of data [91]. The classification was utilized to estimate torque and drag using attributes including weight on bit, rotating velocity, standpipe tension, hook load, and penetration rate. The assessment measures used were the correlation coefficient (R) and average absolute error percentage (AAPE). Based on the study, the recommended approach predicted torque and drag during drilling operations more correctly, and the RF model outperformed the other two models. Next, Kumar and Hassanzadeh’s [92] work focused on the temporal elements of reservoir modeling utilizing a 2D STARS simulation. The study’s goal was to forecast the efficacy of shale barriers in the context of reservoir dynamics, and the ML technique used was RF. The dataset included 240 samples, including predictor factors such as effective formation compressibility, volumetric heat capacity, and thermal conductivity for rock, water, oil, and gas. The assessment measures used were R^2^ and RMSE, with RF indicating effectiveness. The author offered enhancements to the proposed technique by including more training data and features, highlighting the prospect of improving the model’s prediction performance with a larger dataset and more relevant characteristics.

In addition, Ma et al. [93] completed a non-temporal analysis to forecast burst pressure in full-scale corroded O&G pipelines. The study utilized RF, XGBoost, SVM, and LGBM. The dataset included 314 samples with predictor factors such as depth, length, breadth, wall thickness, pipe diameter, steel grade, and burst pressure. The assessment measures employed were R^2^, RMSE, MAE, and MAPE. XGBoost achieved an R^2^ of 99% in training and 98% in testing. The data suggested that the hybrid proposed model, presumably a blend of two models, attained much higher levels. The research by Canonaco et al. [94] performed classification aimed at predicting internal corrosion, considering variables such as odometry, latitude, longitude, elevation, length, flow regime, pressure, mass flow rates, velocity, shear stress, and temperature on a pipeline dataset including 1,700 samples with geometrical and fluid dynamical variables related to pipeline infrastructures. A non-temporal analysis was performed on pipeline data using ML models, specifically XGBoost, SVM, and Neural Networks (NNs). XGBoost achieved an accuracy of 62%. The study suggests that the proposed model’s accuracy needs improvement, indicating the potential for enhancements in accurately predicting internal corrosion in pipeline infrastructures.

Several studies have been conducted on the crude oil domain, such as on corrosion and oil. The researchers used RF and CatBoost to forecast corrosion rates, focusing on non-temporal pipeline and crude oil datasets. It consisted of 3240 samples, including predictors such as stream composition (NO_2_, NH_2_S, and NCO_2_), pressure, velocity, and temperature. The assessment measures used were R^2^, MSE, MAE, and MSE [95]. CatBoost outperformed other models in training and testing, achieving an impressive accuracy of 99.9%. The results reveal that the proposed model is more accurate in estimating corrosion rates for the given pipeline data.

Meanwhile, the other study used the same domain, primarily using data from prior studies on CO_2_–Oil Minimum Miscibility Pressure [96]. The researchers used many ML models, such as XGBoost, CatBoost, LGBM, RF, Deep Multilayer Networks, Deep Belief Networks, and Convolutional Neural Network (CNNs). These 310 samples were included in the collection, which contained data on the N2 and C1 (mole percent of volatile) and CO_2_, H_2_S, and C_2_-C_5_ intermediate crude oil fractions, reservoir temperature, average critical injection temperature of the gas, and molecular weight of the C_5_+ oil fraction. Determining the CO_2_–crude oil system’s lowest miscibility pressure was the goal. CatBoost outperformed the other models, as evidenced by its R^2^ score of 99%. The results demonstrate that the slightest miscibility pressure for the CO_2_–crude oil system can be precisely computed using the suggested model.

A non-temporal analysis of a lithology dataset originating in the Pearl River Mouth Basin was completed in the work by Zhu et al. [97]. An assortment of ML models was employed to classify different lithologies, including Deep Forest (DF), DF + K-means, RF, SVM, and Deep Neural Networks (DNNs). The collection included 601 samples from six classes: limestone, mudstone, sandy mudstone, sandstone, siltstone, and grey siltstone. Based on precision, recall, and Fβ measurements, DF + K-means obtained an accuracy of 90%. The study identified shortcomings in the baseline method, pointing out problems such as noisy data, unsatisfactory minority class prediction, and insufficient labeled data. The findings show the usefulness of DF + K-means in overcoming these issues and improving lithology identification.

The employment of temporal DGA datasets focuses on transformer faults. The researchers used RF and KNN to categorize defect types using the 11,400 sample input parameters [35]. The KNN model attained an accuracy of 88%. Another study was conducted utilizing the same dataset with the employment of a combination of the Gaining-Sharing Knowledge-Based Algorithm (GSK) and XGBoost (GSK-XGBoost) model for the classification [20]. The GSK-XGBoost model scored 50% on accuracy, precision, recall, F1-score, and beta-factor using 128 samples of gas compositions. One of the factors that affected the performance of the model could be the involvement of various gas components and their compositions, such as ammonia, acetaldehyde, acetone, ethylene, ethanol, toluene acetylene, ethylene, ethane, methane, and hydrogen in the DGA dataset. The study discovered an increase in processing time, even after using a devised approach. The proposed model’s accuracy from both studies did not reach 90%. The findings show a trade-off between computing efficiency and accuracy, emphasizing the necessity for a better optimization solution. 

The same DGA processes, considering non-temporal analysis and a classification of fault type, reported an accuracy of 87.06% when using the LGBM [98]. This work’s dataset consisted of 796 samples with gases such as H_2_, CH_4_, C_2_H_2_, C_2_H_4_, and C_2_H_6_. The LGBM outperformed the other ML models, including XGBoost, RF, LR, SVM, NB, the KNN, and DT, for the classification task concerning fault type identification. F1 score, accuracy, precision, and recall were among the evaluation measures for model performance, and the LGBM achieved an accuracy of 87.06%. The study concluded that the model, particularly the LGBM, demonstrated a high level of competence in fault type classification based on the DGA data. However, the enhancement of the model’s accuracy is necessary. 

The non-temporal analysis study by Tewari et al. [8] focused on drilling operations, particularly drill bit selection in Norwegian wells. The researchers used several ML models, including Adaboost, RF, the KNN, NB, MLP, and the SVM. A wide range of drilling-related features were included in the dataset, including 4312 samples with the following characteristics: torque, standpipe pressure, mud weight, real vertical depth, weight on bit, measured dimension, penetration rate, rounds every minute, bit type, bit size, d-exponent, total flow area, mechanical specific energy, depth of cut, and aggressiveness of the drill bit. The primary classification focused on drill bit selection, and the RF model demonstrated an impressive accuracy of 91% in testing and 97% in training. The study’s considerable results show that the proposed method is more stable, accurate, and dependable than the other models used in drill bit selection in Norwegian wells.

The research by Santos et al. [99] employed a temporal exploration centered around well data, specifically focusing on 3W wells. The researcher’s approach involved the application of an RF model for classification, utilizing a dataset encompassing 1984 samples. The dataset included crucial parameters such as the gas lift choke pressure, downstream temperature, and gas lift flow. Their model’s performance was evaluated using metrics like accuracy, faulty-normal accuracy (FNACC), and real faulty-normal accuracy (RFNACC), showcasing an impressive accuracy rate of 94%. The study concludes by emphasizing the efficacy of their proposed method in successfully identifying early faults in the well data. 

The hybrid technique, K-Means+RF, performed admirably with R^2^ values ranging from 92% to 98%, outperforming various baseline approaches in the study, such as using the SVM, Local Outlier Factor (LOF), Local Factor, and RF. The study performed a temporal analysis of reservoir data [100] to cluster sonic (DTC) using the 37 samples from the well log. The features included depth, gamma ray, shallow resistivity, deep resistivity, neutron, density, and CALI. Regarding the temporal analysis of well data from the United States, which has a large field and well-scale, RF was used for clustering barrel of oil equivalent [101]. This experiment used 934 samples, and the features included API, stream date, surface latitude and longitude, formation thickness, TVD, lateral length, total proppant mass, total injected fluid volume, API gravity, porosity, permeability, TOC, Vclay, rate of oil production, gas production, water production, GPI, and frac fluid. Nonetheless, the research brought attention to the necessity of increasing the accuracy since the RF model’s testing and training RMSE values were 17.49% and 7.25%, respectively, suggesting potential overfitting.

The study used various prediction models through temporal research, including LSTM, AdaBoost, LR, SVR, the DNN, RF, and adaptive RF [102], focusing on crude oil data. The employment of adaptive RF in the study shows that the model performed with MAPE, MAE, MSE, RMSE, R^2^, and Explained Variance Score (EVS) values of 112.31%, 52%, 53%, 73%, 99%, and 99%, respectively, outperforming other models. Based on the study’s findings, it’s critical to consider the advantages and disadvantages of the proposed model because it operates for a longer period than other models used in the study. Another study employed RF in their experiment to classify the decommissioning options in the O&G field and utilized 1846 samples from the public O&G dataset [103]. The study was divided into two types of accuracy, with a comparison between RF, KNNs, NB, DT, and NNs. The higher accuracies gathered from RF for full and redundant features that were removed were 80.06% and 80.66%, respectively. However, the suggested approach must be improved because the accuracy was less than 90%.

Following the non-temporal analysis of well logging data, RF with Analog-to-digital converters was used for clustering, with 100 samples and features, including neutron (CNL), gamma ray (GR), density (DEN), and compressional slowness (DTC) [104]. The study’s RMSE (9%), MAE (6%), MAPE (0.031%), and MSE (86%) values indicate that the clustering task’s accuracy might be improved. Further, using pipeline data with climate change components, the study employed the KNN, Multilayer Perceptron Neural Network, multiclass SVM, and XGBoost model to classify temporal analysis [105]. The features included temperature, humidity, and wind speed from 81 samples. The XGBoost model’s accuracy outperformed other models by 92%, leaving room for additional improvement.

Al-Mudhafar et al. [106] worked on well data using LogitBoost, GB, XGBoost, AdaBoost, and the KNN for classification with lithofacies and a well log dataset of 399 samples, which take into account the following parameters: gamma ray (GR), caliper (CALI), neutron (NEU), sonic transit time (DT), bulk density (DEN), deep resistivity (RES DEP), shallow resistivity (RES SLW), total porosity (PHIT), and water saturation (SW). The XGBoost model performed admirably, surpassing other techniques with a Total Percent Correct (TPC) accuracy measures of 97%. Subsequently, Wen et al.’s [107] study on a non-temporal pipeline dataset used recursive feature elimination and particle swarm optimization-AdaBoost for clustering. The collection included 3986 samples with information about landslide risk and long-distance pipelines and consisted of a few parameters, which were landslide susceptibility area (km^2^), percentage (%), and historical landslides (number). The model attained 90% accuracy during training and 83% accuracy during testing, indicating that the proposed clustering strategy must be improved in terms of accuracy.

In the research from Otchere et al.’s study [106,108], which focuses on analysis in the reservoir domain, specifically using the non-temporal Equinor Volve Field datasets, two models employed Bayesian Optimization with XGBoost (BayesOpt-XGBoost) and XGBoost. The dataset comprised 2853 samples, and the classification task involved DT, GR, NPHI, RT, and RHOB as features, aiming to predict Vshale, porosity, and water saturation (Sw). The evaluation metrics encompassed RMSE and MAE. The BayesOpt-XGBoost model achieved an overall accuracy of 93%, with a precision of 98%, a recall of 86%, and a combined F1 score of 93%. Despite these encouraging outcomes, the research indicates that there may be room for improvement in the model’s performance as the suggested approach may not be reliable enough to forecast every output variable. Lastly, a study in the temporal drilling analysis, which used RF and DT, emphasized the need for data confidentiality [109]. The prediction task used weight on drill string rotation speed, rate of penetration, and pump rate as secret features to forecast rock porosity. The RF model performed exceptionally well, with an accuracy of 99% in training and 90% in testing, demonstrating its durability and dependability in handling sensitive drilling data. The literature on the use of DT, RF, and hybrid models is compiled in Table 4.

### 2.5. Application of Interrelated AI Models

The O&G industry has seen a significant spike in the implementation of AI models for more robust predictive capabilities and better decision-making processes. As a kernel-based ML approach, the SVR algorithm has an excellent non-linear modeling capacity and is frequently employed for predictive analytics O&G [112]. MLR analysis is a method of finding a quantity’s reliance on a set of independent factors that are among the most extensively used and ancient. MLR has several advantages: its interpretability, simplicity, and capacity for varied adjustments over time. Additionally, it permits inference based on homogeneity, normalcy, and the intercorrelation between predictor variables and error *εp* [113]. Expanding on AI applications, Guo et al. [114] ventured into non-temporal gas well data, utilizing MLR, SVR, and GPR to predict gas well parameters. The study used 129 samples of M6COND and M6GAS datasets to cluster the output variable, which is the gas well, from the input parameters, including fluid volume, proppant amount, cluster counts, stage counts, total horizontal lateral length, gas saturation, total organic carbon content, and condensate–gas ratio. GPR emerged as the preferred model based on metrics, including RMSE and R^2^. However, the proposed method needs improvement in accuracy.

By classifying oil, gas, and water from 1968 samples from O&G production in five well reservoirs owned by Saudi Aramco, Ibrahim et al. [115] investigated the temporal prediction of corrosion defect depth in pipelines using parameters like location, contact, permeability average, volume, production, wellhead and bottom-hole pressure, and ratio. The study used a variety of AI models, including XGBoost, the ANN, the RNN, MLR, Polynomial Linear Regression (PLR), SVR, Decision Tree Regression (DTR), and RF Regression (RFR). Evaluation measures, including R^2^, MAE, MSE, and RMSE, revealed that the RNN properly categorized oil, gas, and water at 98%, 87%, and 92%, respectively. The suggested model’s output needs to be improved. In the non-temporal domain of O&G production classification. The researcher employed an MLP, RF, and SVR with a few parameters, such as the impact of transportation interruption, safety, health, environmental and ecological factors, and equipment maintenance, to assess 149,940 input samples and a historical record of pipeline failure [116]. The researchers suggested approaches to produce the best-fitting results and use the least computation time.

The dataset of the non-temporal study of reservoir data had 147 samples, including reservoir temperature, oil composition, and gas composition [117], with the objective variable being the minimal miscibility pressure between CO_2_ and crude oil. The assessment statistic used was MSE. The POLY kernel-based SVM model outperformed other models’ accuracy, as seen by its performance. The data reveal that the SVM model with the POLY kernel is excellent in identifying minimal miscibility pressure based on the supplied reservoir. The other temporal analysis focused on the well study by Marins et al. [22], using various ML models. This included RF, the ANN, LSTM, the Independent Recurrent Neural Network, and CatBoost, along with 1984 samples to classify faults in oil well production, including the involvement of features such as P-PDG, T-TPT, P-TPT, Initial Normal, Steady-state, and transient events. The performance evaluation for the ARN model was accuracy at 96%, recall at 84%, and F-measure at 85%. However, this research noted that the best model was not robust due to misclassifications for undesirable events of type 3 and type 8 fault classifications. This indicates the need for further refinement to enhance the model’s robustness in fault detection and classification for these specific events.

Regarding temporal pipeline analysis with an emphasis on Iranian oil fields, Naserzadeh and Nohegar [118] presented an in-depth study that made use of several SVR models enhanced by GA, PSO, Firefly Algorithm (FA), Bat Algorithm, Cuckoo Optimization Algorithm (COA), Grey Wolf Optimizer (GWO), Harmony Search (HAS), Imperialist Competitive Algorithm (ICA), Shuffled Frog-Leaping Algorithm (SFLA), and Simulated Annealing (SA). The models were used to forecast carbon steel corrosion rates using 340 samples and various characteristics such as pit depths, exposure period, operating pressure, and chemical concentrations. The results showed that the SVR-GA-PSO model outperformed the others exceptionally, with an R^2^ of 99%, RMSE of 0.0099, MSE of 9.84 × 10^−5^, MAE of 0.008, RSE of 0.001, and EVS of 0.955. 

The model used in a study by Yuan et al. [119] were Gradient Boosting DT, Physics-Based Bayesian Linear Regression (PBBLR), Bayesian Linear Regression (BLR), and ANN with the usage of non-temporal pipeline domain. With 728 samples from the Supervisory Control and Data Acquisition (SCADA) system, the models attempted to predict factors such as the original length of mixed oil, transportation distance, diameter, and Reynolds number. Although PBBLR is regarded as a superior method, the assessment metrics, i.e., RMSE, MAE, and R^2^, indicate that the accuracy should be improved. The proposed model could benefit from additional improvements. These collective studies showcase the versatile applications of AI models in addressing crucial challenges within the O&G industry, encompassing diverse aspects such as predicting pipeline corrosion, gas well parameters, natural gas pipeline failures, and O&G production outcomes. Incorporating innovative optimization techniques underscores the industry’s commitment to harnessing advanced technologies for enhanced operational efficiency and robust risk management strategies. Table 5 contains previous research published on interrelated AI models for predictive analytics in the O&G field.

### 2.6. Application of Statistical Models

The statistical model’s behavior is a system simulated mathematically, representing the relationships between one or more parameters. Regression and temporal analysis are two statistical modeling techniques that take advantage of this minimization process. Bivariate time series analysis is different from regression analysis, which uses time as an independent or predictor parameter. On the other hand, a bivariate analysis is carried out on two or more statistically linked variables in regression. Furthermore, the bivariate regression model assumes the independence of each measure. To clarify, the order of the predictor and data pairings is not relevant in bivariate regression. However, time series analysis does identify and make use of time dependency to improve the prediction accuracy or understanding of the underlying physical processes [43]. Therefore, identifying temporal patterns requires a deep understanding of mathematics. Temporal modeling techniques that are commonly employed include autoregressive (AR), moving average (MA), autoregressive moving average (ARMA), autoregressive integrated moving average (ARIMA), and seasonal autoregressive integrated moving average (SARIMA) [120,121]. Several studies have explored diverse approaches in the domain of statistical methods for predictive analytics in the O&G industry.

Liu et al. [122] delved into the application of seasonal autoregressive SARIMA, LSTM, and autoregressive (AR) models. The researcher focused on transformer using DGA dataset consisted of 610 samples, considering parameters like H_2_, CH_4_, C_2_H_4_, C_2_H_6_, CO, CO_2_, and total hydrocarbon (TH) to predict dissolved gas concentrations. The evaluation metric, i.e., the Accuracy Relative Error (ARE), highlighted the SARIMA model’s efficacy in capturing seasonal variations and long-term dependencies within the transformer DGA dataset. Yang et al. [62] extended the exploration of statistical methods in wells, employing LSTM and ARIMA models. Concentrating on the Longmaxi Formation of the Sichuan Basin with 3650 data samples, they used date and daily production data to forecast shale gas production. The evaluation metrics, including MAE, RMSE, and R^2^, demonstrated the effectiveness of LSTM in capturing temporal dependencies and ARIMA in handling time series forecasting tasks. However, the model’s accuracy was 63% and needs improvement. Moreover, Xuemei Li et al. [123] contributed to the field of statistical methods, specifically examining the Grey Model (GM), Fractional Grey Model (FGM), Data Grouping-Based Grey Modeling Method (DGGM), ARIMA, PSO for Grey Model (PSOGM), and PSO-based data grouping grey model with fractional order accumulation (PSO-FDGGM). Their study, focusing on natural gas in China, aimed to predict natural gas production during training. MAPE served as the evaluation metric, with PSO-FDGGM showcasing its effectiveness in optimizing the statistical models for accurate predictions, with the result of MAPE is 3.19%. The model’s performance is noteworthy and reliable. 

Collectively, these studies underscore the diverse applications of statistical methods in predictive analytics for the O&G sector. The SARIMA, LSTM, ARIMA, GM, FGM, DGGM, AR, PSOGM, and PSO-FDGGM are recognized as effective tools for handling temporal dependencies, forecasting production, and optimizing model parameters. The specifics of the data and the nature of the predictive analytics work determine which statistical approaches are best, highlighting the need for a customized strategy in the O&G sector. Table 6 highlights previous studies on a statistical model for predictive analytics modeling in the O&G field.

### 2.7. Alternative ML Models Utilized for Predictive Analytics in the O&G

Several researchers have investigated various methods of developing ML models for predictive analytics in the O&G sector. Rashidi et al. [124] investigated the Multi-Ensemble Learning Machine-Genetic Algorithm, Multi-Ensemble Learning Machine-Particle Swarm Optimization (MELM-PSO), Least Squares Support Vector Machine-Genetic Algorithm (LSSVM-GA), and Least Squares Support Vector Machine-Particle Swarm Optimization (LSSVM-PSO) for non-temporal predictions in crude oils. Their considerations included temperature (T), ratio of gas oil solution (Rs), gas concentration (γg), and oil viscosity (API), with an emphasis on the pressure at the bubble point and oil production volume factor, with 638 samples of data from the crude oil database. The evaluation metrics, including RMSE, highlighted the superiority of the MELM-PSO in optimizing model performance. The hybrid proposed model outperformed the empirical method. The temporal analysis was centered on a gas leakage dataset from the research by Gong et al. [125]. For the classification of estimating gas pipeline leakage, the researchers used a variety of ML models, including the CNN, Linear Support Vector Machine (Linear SVM), Gaussian Support Vector Machine (Gaussian SVM), and a combination model, i.e., SVM+CNN. The study utilized a dataset of 1000 samples of gas types such as methane, ethane, propane, isobutane, butane, helium, nitrogen, hydrogen sulfide, and carbon dioxide. The assessment criteria were accuracy, and the accuracy of SVM was 95.5%. The study noted the model’s excellent performance, claiming that the SVM model stood out for accurately estimating gas pipeline leakage using the available information.

Furthermore, Chung et al. [126] investigated PCA, SVM, and LDA for temporal predictions in oil. Their study utilized real-time oil samples, where the pore size (R) remained constant, and the capillary flow rate (l2/t) was a function of interfacial properties (γLG and θ) and viscosity (μ) to predict oil types and 30 samples from real-time oil samples. The evaluation metric used was accuracy, emphasizing the capability of the SVM to capture the underlying patterns in the temporal dataset, with an accuracy predicted of 90%. In the experiment by Mohamadian et al. [127], the analysis focused on a non-temporal well-log dataset from three drilled wellbores. The researchers employed ML models, specifically Multilayer Perceptron with PSO (MLP-PSO) and Multilayer Perceptron with GA (MLP-GA), for the prediction task involving variables such as depth, compressional wave velocity (Vp), shear wave velocity (Vs), bulk density (ρ), and pressure pore (Pp), with the target being the probable depth of casing collapse. The dataset included 22,323 samples, and the evaluation metrics comprised R^2^ and RMSE. The performance of the proposed method indicates that the accuracy of the MLP-PSO model outperformed that of the other models.

Next, the research by Sabah et al. [128] concentrated on drilling activity utilizing non-temporal data from 305 wells drilled and located in the Marun oil field. The researchers tested several ML models, including the hybridization of the Least Square Support Vector Machine (LSSVM) with COA, PSO, and GA, MLP-COA, MLP-PSO, MLP-GA, LSSVM, and MLP, to predict parameters such as northing, easting, depth, meterage, time of drilling, formation type, size of hole, weight on bit, flow rate, weight of mud, MFVIS, retort solid, pore pressure, fracture pressure, fan 600/fan 300, Gel 10min/Gel 10s, pump pressure, and rpm. The goal variable was the severity of mud loss. The MLP-GA model had an RMSE of 93%, while the suggested model was accurate. Shi et al. [129] used a Hybrid-Physics Guided-Variational Bayesian Spatial-Temporal Neural Network to analyze natural gas across time. The study aimed to forecast natural gas concentrations using a dataset of 600 samples. The predictor variables were geometry size, release point position, release diameter, released gas, volumetric release rate, duration, and sensor placement. The R^2^ value was used as an evaluation metric, and the Hybrid-Physics Guided-Variational Bayesian Spatial-Temporal Neural Network received a score of R^2^ is 99% It can be concluded that the findings imply the Hybrid-Physics Guided-Variational Bayesian Spatial-Temporal Neural Network enhanced the spatiotemporal forecasting performance.

Furthermore, the temporal analysis focused on well data, specifically within the context of 3W wells by Machado et al. [130]. The research involved the application of LSTM and One-Class Support Vector Machine (OCSVM) models for classification, utilizing a dataset comprising 1984 samples. The classification task aimed to identify the following types of faults: P-PDG, P-TPT, T-TPT, P-MON-CKP, and T-JUS-CKP. The evaluation metrics included recall, specificity, and accuracy, with the OCSVM model achieving an accuracy of 91%. The study found that feature selection did not improve classifier accuracy, and the proposed model demonstrated a lack of robustness in effectively classifying the two types of faults in the well data. The temporal analysis of the research by Carvalho et al. [10] focused on well data, specifically 3W wells. The study used ML models such as Ordered Nearest Neighbors, Weighted Nearest Neighbors, LDA, and QDA to perform a classification job with 1984 samples. The classification sought to forecast flow instability by detecting events like P-PDG, P-TPT, T-TPT, P-MON-CKP, T-JUS-CKP, and CLASS. The evaluation measures included recall, specificity, and accuracy, with the ONN reaching an accuracy of 81%. However, the study’s author recommended looking into different metaheuristic methodologies, indicating a possibility for better performance in forecasting flow instability from the well data.

In the study by Zhou et al. [131], the analysis in the reservoir domain was conducted with DT and SVM models on high-resolution non-temporal Formation Micro-Imager (FMI) data. The classification task aimed to categorize how logging units react to sedimentary pyroclastic rock, regular pyroclastic rock, and pyroclastic lava for lithologically classifying pyroclastic rocks. The SVM’s model had an impressive accuracy of 98.6%, surpassing the threshold of 95%. The study emphasized the efficacy of the suggested model in lithologic classification by highlighting its significantly superior performance. In Zhang et al.’s [132] study, which involved a temporal analysis in the pipeline domain, CNN, SVM, and SVM+CNN models were applied to a leakage dataset containing 1000 samples. The prediction task focused on length, outer diameter, wall thickness, and location in the model to predict leakage in tight sandstone reservoirs. The SVMCNN model achieved a high accuracy of 95.5%, outperforming other methods. This highlights the advantages of the suggested methodology over other methods for anticipating leaks in tight sandstone reservoirs. Collectively, these studies highlight the application of alternative ML models, specifically SVM and MLP, in addressing various predictive analytics challenges in the O&G industry. The selection of the model depends on the nature of the data and specific predictive task at hand, showcasing the versatility and effectiveness of these models in optimizing predictions for different parameters and scenarios. 

Zuo et al. [133] addressed natural gas leakage in SCADA data using a network and OCSVM hybrid with a few other ML models, including Basic Autoencoder (BAE), Convolutional Autoencoder (CAE), LSTM with Autoencoder (AE), RF, PCA, Variational Autoencoders (VAE), and LSTM-AE- isolation forest (IF), with 9980 samples of input data, to demonstrate the efficiency of DL models for managing complicated and time-varying gas data to ensure precise categorization. The proposed model, i.e., LSTM- AE-OCSVM, had a greater accuracy of 98%, and the researcher proposed using anomalous data in future studies. Meanwhile, Martinez and Rocha [67] focused on reservoirs and used 3,257 samples from the Volve and UNISIM-IIH oil fields to examine LSTM and GRU models. With an impressive R^2^ of 99%, the GRU model demonstrated its superiority in O&G forecasting when classifying oil, gas, water, or pressure. Within the field of reservoir clustering, Chen et al. [134] applied K-Means Clustering and KNN models to a range of shale reservoirs, including Antrim, Barnett, Eager Ford, Woodford, Fayetteville, Haynesville, and Marcellus. With 55,623 samples involving well location, depth, length, and production starting year, the K-MC model outperformed the alternative models, with an R^2^ of 0.18. To classify wells using the 3W oil well dataset, Fernandes et al. [135] investigated models like OCSVM, LOF, Elliptical Envelope, and AE using feedforward and LSTM. The LOF model showed an F1 score of 85%, with an emphasis on fault identification utilizing parameters like P-PDG and T-JUS-CKGL. Although deemed acceptable, the accuracy of the suggested approach can be increased.

In the domain of non-temporal well analysis in the oil fields in the Middle East, Gao et al. [136] utilized the group method of data handling (GS-GMDH) models with 2748 samples. The researchers predicted pore pressure based on various parameters such as gamma ray (spectral) (SGR), density (RHOB), gamma ray (corrected) (CGR), and sonic transit time (DT). The GS-GMDH model exhibited an RMSE of 1.88 psi and an R^2^ of 0.9997, showcasing higher accuracy. Using geological data from 180 samples, Cirac et al. [137] investigated a few models, including RF, Gradient Boosting Regressor, Bagging, CNN, KNN, and Deep Hierarchical Decomposition models, in their investigation of temporal reservoir analysis. They aimed to classify a variety of parameters, including porosity, fracture porosity, fracture permeability, rock type, net gross, matrix permeability, water relative permeability, formation volume factor, rock compressibility, pressure dependence of water viscosity, gas density, water density, vertical continuity, relative permeability curves, oil–water contact, and fluid viscosity. The Deep Hierarchical Decomposition model decreased computing speed, with an MAE for oil production of 0.76%. Within the framework of gas analysis, Dayev et al. [138] employed the M5P tree model and RF, Random Tree, Reduced Error Pruning Tree (REPT), GPR, SVM, and Multivariate Adaptive Regression Spline (MARS) models with 201 samples from a Coriolis flow meter. They aimed to classify wet gas flow rate (kg/h) and absolute gas humidity (g/m^3^) for the estimation of dry gas flow rate (kg/h). The GPR-RBKF model outperformed other models, with an MAE of 163.3266 kg/h and an RMSE of 483.1359 kg/h. Table 7 summarizes previous works on the application of ML models for predictive analytics modeling in O&G fields.

## 3. Literature Review Assessment

Analyzing and evaluating the existing literature is crucial for survey research as it provides readers with an in-depth discussion that will be helpful. Considering the previously reported review of ML-based models for predictive analytics modeling for O&G fields, this section summarizes and discusses numerous key points.

**Figure 4:**
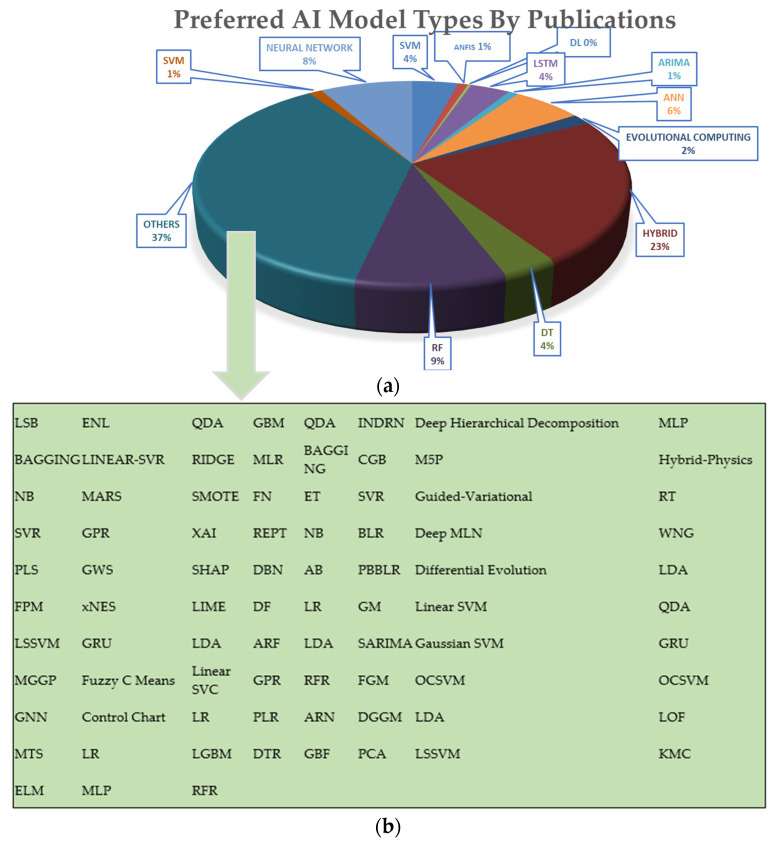
Preferred AI model types in the research articles about predictive analytics in the O&G field: (**a**) overview of the AI models used in the publications and (**b**) extended “others” section.

**Figure 5:**
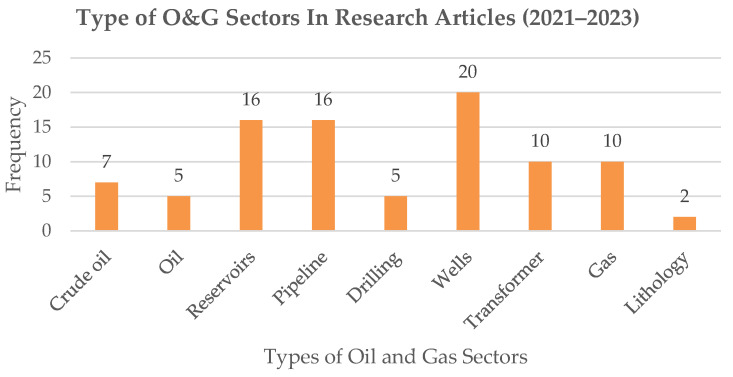
Types of O&G sectors in research articles from 2021 to 2023.

**Figure 6:**
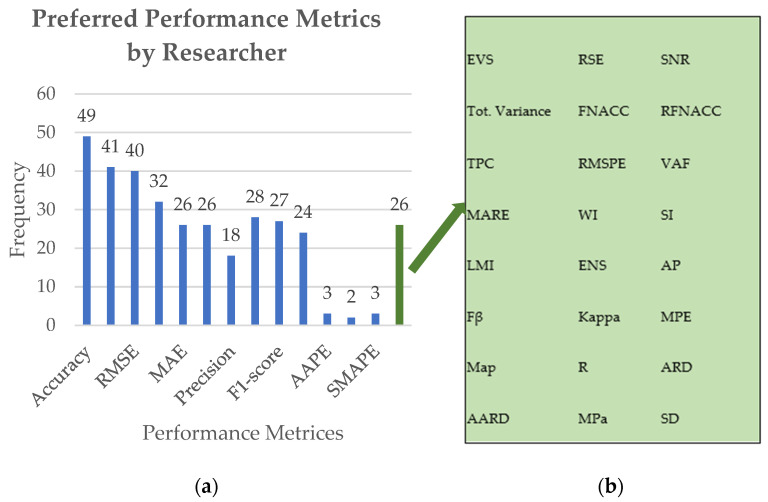
Preferred performance metrics by the researcher: (**a**) combination of performance metrics used in publications. (**b**) All additional performance metrics displayed.

**Figure 7:**
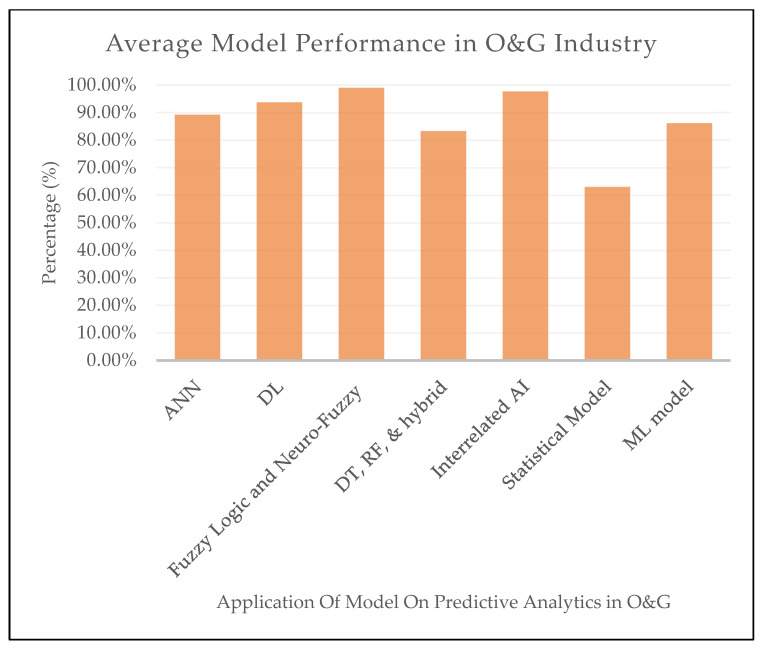
Average accuracy of ML models in the O&G industry.

- Table 1, Table 2, Table 3, Table 4, Table 5, Table 6 and Table 7 provide a comprehensive overview of the reviewed papers, presenting essential details such as the author names, applied AI model types, temporality of the dataset, domain of the O&G model in the study, dataset sources, number of data samples, parameters for input and output, measures for the performance employed, best models found, and advantages or drawbacks of the performing models. The researchers consistently focused on carefully selecting input combinations for O&G predictive analytics modeling.
- ANN models can be expanded from binary to multiclass cases. Furthermore, the complexity of ANN models may be easily changed by modifying model structure and learning methods and assigning transfer functions using empirical evidence or correlation analysis. The findings revealed that ANNs could effectively predict, classify, or cluster O&G cases, including crater width in buried gas pipelines, corrosion defect depth, flowing bottom-hole pressure in vertical oil wells, concentrations of gas-phase pollutants for contamination removal, drilling-related occurrences based on epochs, age, formation, lithology, and fields, as well as predicting gas routes and chimneys in drilling activities and DGA datasets. ANNs may be compared to various models, like the SARIMA and QDA.
- Reviewed articles from 2021 to 2023: RF has become much more popular in the predictive analytics for O&G than other modeling techniques, like the MLP, DT, and LSTM, because it prevents overfitting and is more accurate in prediction. In the O&G sector, RF appears to be a typical, flexible, and effective ML framework because of its capacity to handle complicated O&G datasets that may be fragmented. The O&G industry has become another field with data scarcity for modeling. In pipeline failure risk prediction and transformer fault classification, RF is included in model ensembles to help achieve good results. Its use in drilling, well data analysis, lithology identification, crude oil data analysis, and burst pressure prediction demonstrates RF’s robust application performance. RF stands out for its dependability, obtaining excellent accuracy, precision, and recall values in many applications within the O&G area, emphasizing its applicability for multiple data formats such as binary or multiclass cases.
- The O&G industry has seen a rise in the use of DL, an effective subset of ML, especially for predicting the lifespan of equipment and modeling groundwater levels. DL frameworks, especially the CNN and LSTM, outperform other models in prediction accuracy. Industry uses of DL include assessing algorithm performance, integrating data into DL algorithms, and developing simulation frameworks. Significant studies demonstrate DL’s efficacy in estimating oil output and pressure in wells, identifying pipeline fractures, and producing hydrocarbons in the gas sector. The evaluations of hybrid models, such as DCNN+LSTM and LSTM+Seq2Seq, show outstanding accuracy, indicating DL’s potential for optimizing operations and decision-making processes in the O&G field. The hybrid model is more efficient due to feature extraction and the capacity to learn patterns in extended data sequences.
- AI models are widely employed in the O&G sector to deliver predictive analytics. In non-linear modeling, SVR is a kernel-based ML method often used to translate data to a higher-dimensional space. This makes it an effective tool for regression problems with complicated input and interaction of target variables. MLR is still an excellent approach for examining dependencies since it is a powerful tool for analyzing the connection between dependent and several independent variables. Non-temporal gas well data are analyzed using MLR, SVR, and GPR models because they provide a good blend of interpretability, simplicity, performance, and adaptability. However, the decision between these models is ultimately determined by the dataset’s particular properties and the problem’s needs. The other research focused on the temporal prediction of corrosion in pipes using several AI models, with the RNN showing promising results. Non-temporal O&G production categorization, reservoir data analysis, and transformer fault prediction were all explored using various AI models, demonstrating industry flexibility.
- The O&G sector replicates real-world system behavior with mathematical models, namely regression and time series analysis. Statistical models such as the SARIMA, AR, and ARIMA are more accurate since they account for temporal relationships. Research has validated the efficacy of the SARIMA in forecasting DGA gas concentrations in transformers, highlighting its ability to capture seasonal fluctuations based on each temporal data point. These techniques forecast shale gas output, producing a satisfactory mean outcome. It has been proven that statistical approaches are adaptable to dealing with temporal dependencies and forecasting concerns in the O&G area.
- The limited sample size of the dataset utilized in earlier research on predictive analytics in O&G industries is a key limitation that can have a major impact on the results’ generalizability and dependability. It is challenging to obtain reliable results from small sample numbers since they frequently result in more variability and fewer accurate estimations. This limitation may also lead to a loss of statistical power, which lowers the capacity to identify important variations or connections in the data. Additionally, there is a higher chance that a smaller sample size of data may not accurately reflect the larger population, which could introduce bias and restrict the findings’ application to other groups. Therefore, to maintain robustness and accuracy, researchers need to take precautions when interpreting studies based on limited datasets and think about confirming their findings using larger and more varied sample sizes.
- A few input parameters were used to detect defects in wells utilizing various sensors in predictive analytics including classified, clustered, and forecasted. Because of the data’s accessibility and availability, researchers regularly employ P-PDG, P-PDG, P-TPT, T-TPT, and P-MON-CKP (five parameters) as input parameters. Data limitations are widespread due to the difficulty of digging wells in severe environments such as the deep sea. However, there are two types of models implemented RF model in the previous study. Between RF model used 15 input parameters and the RF model used five parameters then the performance results of those two models are compared. The outcomes of employing the 15 input parameters with the DT model were superior to the five input parameter models. Table 8 outlines the input parameters utilized by the researchers in their research papers.
- Detecting internal transformer failures is another O&G-related topic that has been the subject of several previous studies. Specifically, a few gas compositions were used as input variables, including acetylene (C_2_H_2_), ethylene (C_2_H_4_), ethane (C_2_H_6_), methane (CH_4_), and hydrogen (H_2_), which were mainly applied across the studies because of the high correlation between the input variables and the target variables in detecting the fault in the transformer. However, the detection of other parameters such as total hydrocarbon (TH), carbon monoxide (CO), carbon dioxide (CO_2_), ammonia (NH_3_), acetaldehyde (CH_3_CHO), acetone (CH_32_CO), toluene (C_6_H_5_CH_3_), oxygen (O_2_), nitrogen (N_2_), and ethanol (CH_3_CH_2_OH) varied between studies. These parameters were chosen because of the weak correlation ranking between the input and target variables; so, not all the studies implemented the gas compositions mentioned earlier. A few input variables, including C_2_H_2_, C_2_H_4_, C_2_H_6_, CH_4_, and H_2_ (five variables), were included in the study article’s model comparison. The results showed that models like KNN, QDA, and LGBM had accuracies of 88%, 99.29%, and 87.06%, respectively. In contrast, the accuracies of the MTGNN, KNN+SMOTE, and RF, with accuracies of 92%, 98%, and 96.2%, respectively, were obtained when the models employed C_2_H_2_, C_2_H_4_, C_2_H_6_, CH_4_, H_2_, TH, CO, CO_2_, NH_3_, CH_3_CHO, CH_32_CO, C_6_H_5_CH_3_, O_2_, N_2_, and CH_3_CH_2_OH (15 variables) in their research. As can be observed from the average accuracies, the use of 15 variables produces superior outcomes than the five variable models. Previous research publications may be found in Table 9.
- Table 10 summarizes the input parameters for a well logging predictive analytics model. The researchers commonly used 14 parameters for well logging, including gamma ray (GR), sonic (Vp), deep and shallow resistivities (LLD and LLS), neuro-porosity (NPHI), density (RHOB), caliper (CALI), neutron (NEU), sonic transit time (DT), bulk density (DEN), deep resistivity (RD), true resistivity (RT), shallow resistivity (RES SLW), total porosity (PHIT), and water saturation (SW). The correlation coefficient between the input parameters and the target variables is essential to determine which parameters are appropriate for predictive analytics and the data type, which may be numerical or categorical. Thus, a few important variables can be chosen to construct the best model for increased accuracy. However, the model using 14 variables produced a substantial result of 97% by including XGBoost in their research, but the study that only utilized GR, Vp, LLD and LLS, NPHI, and RHOB and used the LSTM model achieved a slightly lower result of 94%. These three well-known datasets, which have been utilized in recent research in the O&G sector, demonstrate the importance of determining the correlation between target and input parameters to compare which variables are appropriate for models to provide significant outcomes in the research.
- The assessment of O&G research revealed an increase in published papers over time. As seen in Figure 2, the rise in O&G discoveries due to the dependence of technological advancements on the usage of gas and petroleum, as well as the annual progress of ML and AI tools, has resulted in more studies in this field utilizing AI-based models. As shown in Figure 2, there was an increase in growth throughout 2021, with 32 research publications published in this field. However, the number of articles released in 2022 decreased by seven, with just 25 published research papers. This reduction can be attributed to the continued development of AI and the gradual progression of interest in O&G research. It exhibits a positive trend, with 34 articles published in this field by 2023. This increase may be impacted by recognizing the necessity for improvement in the AI-based model in the O&G area. Many O&G companies have followed the IR4.0 road to integrate AI in their organization and reduce the likelihood of future expense utilization by forecasting future events.
- Throughout the research period, developments in AI models resulted in more complicated and interconnected models, giving researchers tools to construct more exact and resilient models. A similar finding was reached while investigating the use of various models in predictive analytics in the O&G industry during the last three years. Figure 4a depicts a thorough breakdown of the most common model types used for predictive analytics in the O&G industry, illustrated by a pie chart. The chart shows that the most widely used models, there is 37% out of all models are classified as “others”, which primarily include foundational models such as SVR, GRU, MLP, and boosting-based models (shown in Figure 4b). Due to their improved efficiency, accuracy, and capacity to handle non-linear datasets, these models have become quite popular. This selection of models shows that there is still a lot of remaining potential in this field.
- The analysis of predictive analytics research publications from 2021 to 2023 focuses heavily on several areas of the O&G sector. Crude oils (7), oil (5), reservoirs (16), pipelines (16), drilling (5), wells (20), transformers (10), gas (10), and lithology (2) all appear as similar subjects in different research. The frequency of these terms demonstrates the industry’s strong interest in using predictive analytics to optimize operations and decision-making in various sectors, including reservoir management, drilling procedures, pipeline integrity, and transformer health. This trend represents a deliberate effort in the O&G industry to use sophisticated analytics for greater efficiency, risk management, and overall operational excellence. Figure 5 is the graphical summary of the types of O&G sectors in research articles.
- Several performance measures have been utilized in O&G research, demonstrating diverse assessment criteria for predictive analytics models (see Figure 6). The performance metrics help understand the models’ performance since they might show many model characteristics. Figure 6a, which shows the various performance measures used in the research, demonstrates that accuracy (49) was the most preferred for calculating the correctly predicted value versus the actual one. This performance measure is appropriate for categorical data types and classification predictive analysis because it is simple to grasp and indicates whether all the classes are balanced. However, utilizing accuracy for unbalanced classes has limitations since it can be deceptive; alternative measures like precision, recall, F1 score, or AUC may be more helpful. Aside from that, the researchers’ second chosen performance indicator in their research is R^2^ (41). This performance indicator is commonly employed in regression analysis and numerical data since it measures the relationship between the independent and dependent variables.
- Furthermore, R^2^ is simple to read because it ranges from 0 to 1, with closer results to 1 indicating perfect variability between independent and dependent variables. However, there is a disadvantage to using only R^2^ to demonstrate how effectively the model reacts. One of the disadvantages is that it is vulnerable to outliers; even a single outlier might alter the results. Figure 6b is an expansion of the “others” section that depicts the additional performance indicators used in the previous studies.
- Based on the data presented in Table 11, a thorough analysis of model performance for diverse applications identifies numerous key performers across multiple categories. In the field of ANNs, significant high performers include ANN models with accuracies of 99.6% and ANNs integrated with PSO (ANN+PSO) with 99% accuracy. This suggests that adding optimization techniques such as PSO can considerably improve ANN performance. DL models also perform well, with DCNN+LSTM obtaining 99.37% accuracy and GRU models reaching 99% accuracy. These studies demonstrate the effectiveness of DL systems, particularly in managing complicated data patterns.
- Within the class of Fuzzy Logic and Neuro-fuzzy models, every variation—LSSVM+CSA, ANFIS+PCA, and Control Chart+RF—achieves 99% accuracy on average. This consistency emphasizes the dependability of Fuzzy Logic systems in certain applications. DT, RF, and hybrid models exhibit considerable variability, with top performers such as DT and CATBOOST reaching 99.9% accuracy. However, the high number of models with much lower accuracies indicates a considerable sensitivity to certain data properties and model settings.
- Interrelated AI models, particularly the SVR combined with the Genetic Algorithm and Particle Swarm Optimization (SVR+GA+PSO), outperform others with 99% accuracy, demonstrating the potential of hybrid approaches to increase prediction accuracy. The ARIMA is the most accurate statistical models in the research, with a performance of 63%. However, it has limitations when dealing with complex datasets compared to advanced AI models.
- Finally, in predictive analytics for the O&G domain, the Hybrid-Physics Guided-Variational Bayesian Spatial-Temporal Neural Network and GRU models approach 99% accuracy, demonstrating the usefulness of merging domain-specific knowledge with sophisticated neural network designs. ANN and DL models perform well in a variety of situations, but using hybrid approaches and optimization techniques can improve their accuracy even more. However, the difference in performance across DT and RF models indicates that careful model selection and tuning are necessary to achieve optimal outcomes.
- The study indicates various patterns in model performance. ANNs have few outliers of the model’s performance but show excellent accuracy for the MLP, for example, has 10% accuracy. While there is significant volatility in the model’s performance, DL models consistently perform well, as seen by Faster R-CNN+ClusterRPN’s 71% accuracy. Fuzzy Logic models provide particularly consistent high performance. DT and RF models are very variable, with some obtaining outstanding accuracy and others doing poorly. Interrelated AI models have consistently obtained excellent accuracy. Statistical models, such as the ARIMA, perform poorly compared to other categories, showing their limits with complicated datasets. Predictive analytics models normally perform well. Yet, there is a significant outlier in predictive analytics modeling. For example, K+MC with 18% accuracy.
- Performance levels differ among model categories, as shown in Figure 7. ANN models perform well on average, with an accuracy of 89.23%, but performance can vary greatly depending on specific variations and modifications, as shown by several outliers. DL models perform well, with an average accuracy of 93.73%, demonstrating less variability and solid outcomes across diverse versions. Fuzzy Logic and Neuro-fuzzy models stand out for their excellent and constant performance, with an average accuracy of 99%, making them extremely trustworthy for their applications. DT, RF, and hybrid models exhibit great variability; although models like CATBOOST and DT attain excellent accuracy, others, such as RF+Analog-to-digital converters, perform poorly. Interrelated AI models perform consistently well, with an average accuracy of 97.67%. In comparison, the ARIMA model from the statistical model category performs inadequately, with 63% accuracy, demonstrating limits in dealing with complex information. Models used for predictive analytics in the O&G field typically perform well, although there are a few distinct instances. Overall, while the most advanced AI models perform well, the diversity in particular categories emphasize the significance of model selection and modification for the best outcomes.

## 4. Future Research Directions

As predictive analytics in the O&G industry continues to evolve, several avenues for future research and development emerge. First, exploring the integration of advanced Deep Learning techniques, such as RNN and LSTM networks, could enhance the temporal predictive capabilities of existing models. These architectures are adept at capturing sequential dependencies and time series patterns, which could prove invaluable for forecasting dynamic aspects like O&G production rates or pipeline conditions. Second, investigating explainability and interpretability in complex models, such as ensemble techniques and Deep Learning networks, continues to be an important area of research. Developing methods to elucidate the decision-making processes of these models can enhance the trust and acceptance of predictive analytics in decision support systems within the O&G domain.

Furthermore, there is potential for extending research into the optimization of hybrid models, focusing on refining parameter-tuning strategies and evaluating the robustness of these approaches across diverse datasets and scenarios. For instance, understanding how QPSO or FDGGM parameters impact model performance could lead to more effective and efficient hybrid predictive systems. Additionally, exploring predictive analytics for emerging challenges in the industry, such as sustainability, environmental impact, and safety, could open new avenues for research. Predicting the environmental consequences of O&G activities or developing models for proactive safety monitoring could contribute significantly to the industry’s responsible and sustainable practices.

Finally, comprehensive benchmarking studies are needed to compare the performance of various predictive models under many circumstances and datasets. This could facilitate the identification of the most suitable models for specific applications within the O&G sector, providing practitioners with insightful information for making decisions. In conclusion, future research in predictive analytics for the O&G industry should delve into advanced Deep Learning architectures, enhance model interpretability, optimize hybrid approaches, address emerging challenges, and conduct systematic benchmarking studies to advance the state-of-the-art methods in this critical domain.

## 5. Conclusions

This review aimed to provide a thorough overview of the utilization of ML models in simulating predictive analytics within the O&G sectors. From 2021 to 2023, we collected data from respectable journals indexed in Web of Science, Science Direct, Scopus, and IEEE. The analysis revealed that seven iterations of ML models had been employed in predictive analytics modeling for the O&G industry. The survey identified key components within existing predictive analytics models for the O&G field, encompassing Key elements of current predictive analytics models for the oil and gas industry were identified by the survey. These elements included model types, temporal aspects of the data and the field, the name of the data, dataset types, predictive analytics methodologies (such as classification, clustering, or prediction), model input and output parameters, performance metrics, optimal models, and the advantages and disadvantages of the models. Rigorous scientific assessments and evaluations were conducted on the surveyed studies, leading to detailed discussions on numerous findings. This review also highlights various potential future research directions based on the current state of the literature, providing insightful information to interested professionals in this sector.

## Figures and Tables

**Table 1:** A list of research articles on predictive analytics in the O&G field using ANN models.

Reference	Models	Temporality	Field	Dataset	Class	Input Parameter	Output Parameter	Performance Metrics	Best Model	Advantages/Disadvantages
[46]	SVM, QPSO-ANN, WQPSO-ANN, and LWQPSO-ANN	Non-temporal	Pipeline	Buried gas pipeline 99 samples	Prediction	Pipe diameter (mm), operating pressure (MPa), cover depth (m), and crater width (m)	Crater width	Map, R^2^, MSE. RMSE, MAPE, and MAE	LWQPSO-ANN	The proposed method outperformed the other method by more than 95%.
[48]	RF, KNN, and ANN	Non-temporal	Wells	Middle East fields: for vertical wells 206 samples	Prediction	Oil gravity (API), well perforation depth (depth (ft), surface temperature (ST (F)), well bottom-hole temperature (BT (F)), flowing gas rate (Qg (Mscf/day)), flowing water rate (Qw (bbl/day)), production tubing internal diameter (ID (inches)), and wellhead pressure (Pwh (psia)).	Vertical oil wells’ flowing bottom-hole pressure Pwf (psia)	MSE and R^2^	ANN R^2^ = 97% (training) and 93% (testing)	The suggested model had a much greater value than the other models.
[49]	ANN, LSB, and Bagging	Non-temporal	Oil	Oil shale 2600 samples	Prediction	Air molar flowrate, illite silica, carbon, hydrogen content, feed preheater temp, and air preheater temp	Petroleum output with CO_2_ emissions	RMSE	ANN RMSE oil yield = 99.6% RMSE CO = 99.9%	The suggested model’s precision outperformed the performance of the remaining models.
[50]	NB, KNN, DT, RF, SVM, and ANN	Temporal	Oil	Ocean slick signature 769 samples	Classification	The data are confidential.	Sea-surface petroleum signatures	Accuracy, sensitivity, specificity, and predictive values	ANN Accuracy = 90%	The proposed model did not give significant results.
[47]	ANN, SVM, EL, and SVR	Non-temporal	Pipeline	The data are confidential.	Classification	CO_2_, temperature, pH, liquid velocity, pressure, stress, glycol concentration, H_2_S, organic acid, oil type, water chemistry, and hydraulic diameter	Corrosion defect depth	MSE and R^2^	EL, ANN, and SVR	The proposed methods had a low error rate.
[51]	PLS, DNN, FPM, FP-DNN, and FP-PLS	Non-temporal	Pipeline	Long-distance pipelines 2093 samples	Prediction	Mixed oil length, inner diameter, pipeline width, Reynolds number, equivalent length, and actual mixed oil length.	Mixed oil length	RMSE	DNN RMSE = 146%	The error rate is not convincing and is the highest one.
[52]	ANN and GA	Non-temporal	Crude Oil	ASPEN HYSYS V11 process simulator	Prediction	Well, feed flow rate, the pressure of gas products, interstage gas discharge pressure, isentropic efficiency of centrifugal compressor	Enhance petroleum production	R^2^	ANN	The performance of ANN+GA to enhance petroleum production is improved.
[53]	ANN	Non-temporal	Gas	The data are confidential. 104 samples	Prediction	Sulfur dioxide, methanol, and α-pinene	The removal of gas-phase M, P, and H in an OLP-BTF and a TLP-BTF.	R^2^ and MSE	ANN+PSO R^2^ > 99%	The proposed model is good, and the author suggested improving the model with real-world applications.
[54]	ANN, LSSVM, and MGGP	Temporal	Reservoir	Previous experimental and simulation studies 223 samples	Prediction	Height, dip angle, wetting phase viscosity, non-wetting phase viscosity, wetting phase density, non-wetting phase density, matrix porosity, fracture porosity, matrix permeability, fracture permeability, injection rate, production time, and recovery factor	Gas-assisted gravity drainage (GAGD)	R^2^, RMSE, MSE, ARE, and AARE	ANN R^2^ = 97% RMSE = 0.0520	The ANN outperformed the proposed method (MGGP = 89% (R^2^) and 0.0846 (RMSE)).
[59]	GNN and Multivariate Time Series	Temporal	Transformer	DGA 1408 samples	Clustering	H_2_, CH_4_, C_2_H_6_, C_2_H_4_, C_2_H_2_, CO, and CO_2_	Power transformer fault diagnosis	Accuracy	MTGNN Accuracy = 92%	The model was proven to be effective in its application.
[33]	ANN and Multilayer Perceptron with Backpropagation	Non-temporal	Crude Oil	Recent literature 172 samples	Prediction	Pressure (P) [Kpa], temperature (T) [C], liquid viscosity (uL) [c.p.], gas viscosity (uG) [c.p.], liquid molar volume (VL) [m^3^/kmol], gas molar volume (VG) [m^3^/kmol], liquid molecular weight (MWL) [kg/kmol], gas molecular weight (MWG) [kg/kmol], and interfacial tension (o) [Dyne]	Diffusion coefficient (D) [m^2^/s]	MSE and RMSE	Multilayer Perceptron with Backpropagation R^2^: Training dataset = 88% Testing dataset = 89%	The suggested model had low accuracy. The hybrid model did not improve the model’s accuracy.
[55]	GA with a backpropagation neural network	Temporal	Crude oil	Crude oil gathering and transportation system 509 samples	Prediction	The inlet temperature of the combined system, outlet temperature of the combined system, inlet pressure of the combined system, outlet pressure of the combined system, inlet and outlet temperature of the transfer station system, inlet and outlet pressure of the transfer station system, inlet and outlet of the oil gathering wellhead system, treatment liquid volume, total power consumption, and total gas consumption	Energy = 99% Heat = 99% Power = 97%	R^2^	GA with a backpropagation neural network	The model provided considerable results.
[56]	MLP and ANN	Temporal	Drilling	Egyptian General Petroleum Corporation (EGPC) 1045 samples	Clustering and classification	Epoch, age, formation, lithology, and fields	Gas channels and chimney prediction	RMSPE	MLP RMSE = 0.10	The proposed model had a lower error rate and outperformed the other method.
[57]	ELM, Elastic Net Linear, Linear-SVR, Multivariate Adaptive Regression Spline, Artificial Bee Colony, PSO, Differential Evolution, Simple Genetic Algorithm, GWO, and xNES	Temporal	Shale gas	YuDong-Nan shale gas field	Prediction	The minerals were quartz, calcite, dolomite, barite, pyrite, siderite, clay, and K-feldspar.	Total organic carbon	R^2^, RMSE, MAE, MAPE, MARE, and WI	DE+ELM = 0.497 (RMSE)	Acceptable results for hybrid ELM models with the proposed method, except for GWO
[58]	MLP and Radial Basis Function Neural Network	Temporal	Reservoir	Gullfaks in the North Sea	Prediction	Injection rate for water, gas, and half-cycle time. Downtime.	Water alternating gas	Average absolute relative deviation (AARD)	MLP-LMA	The proposed model outperformed the other two proxy models and significantly reduced the simulation time.

**Table 2:** Summary of the published research on Deep Learning models for predictive analytics in the O&G field.

Reference	Models	Temporality	Field	Dataset	Class	Input Parameter	Output Parameter	Performance Metrics	Best Model	Advantages/Disadvantages
[63]	LSTM and GRU	Temporal	Reservoir	Metro Interstate Traffic Volume dataset, Appliances Energy Prediction dataset, and UNISIM-II-M-CO 301 samples	Prediction	Fluid production (oil, gas, and water), pressure (bottom-hole), and their ratios (water cut, gas–oil ratio, and gas–liquid ratio).	Oil production and pressure	MAE, RMSE, and SMAPE	LSTM + Seq2Seq and GRU2 architectures	The author suggested looking at another metaheuristic method, such as GA.
[61]	DCNN + LSTM, ANN, SVR, LSTM, and RNN	Temporal	Pipeline	Real-time pipeline crack 90,000 data samples	Prediction	Pipeline condition, label, crack size, data length, sampling frequency, and tube pressure	Natural gas pipeline crack	RMSE, MAPE, MAE, MSE, and SNR	Optimized DCNN + LSTM Accuracy = 99.37%	The model showcased impressive performance.
[64]	LSTM, Bi-LSTM, and GRU	Temporal	Well	West Natuna Basin dataset 11,497 samples	Prediction	GR, Vp, LLD, LLS, NPHI, and RHOB	Well log data imputation	MAE, RMSE, MAPE, and R^2^	LSTM RMSE = 94%	The suggested model provided a greater accuracy.
[65]	KNN, SVM, and XGBoost	Non-temporal	Transformer	DGA local power utilities and IEC TC 10 dataset 1530 samples	Classification	F7, F10, F17, F18, F19, F21, F24, F34, F36, and F40	Transformer faults	Accuracy, precision, and recall	KNN + SMOTE Accuracy: DGA = 98% IEC TC 10 = 97%	The proposed model outperformed the other model.
[66]	DL, DT, RF, ANN, and SVR	Non-temporal	Reservoir	Sorush oil field and oil field in southern Iran 7245 samples	Prediction	Measure choke size (D64), wellhead pressure (Pwh), oil specific gravity (γo), and gas–liquid ratio (GLR).	Wellhead choke flow rates	RMSE and R^2^	DL R^2^ = 99%	Compared to the other model, the accuracy of the suggested model was greater.
[67]	LSTM and GRU	Temporal	Reservoirs	UNISIM-IIH and Volve Oilfield 3257 samples	Classification	Oil, gas, water, or pressure	Oil and gas forecasting	SMAPE and R^2^	GRU R^2^ = 99%	The proposed model had the highest accuracy.
[68]	Faster R-CNN_Res50, Faster R-CNN_Res50_DC, Faster-R_CNN_Res50_FPN with Edge Detection, and Cluster+Soft-NMS	Non-temporal	Well	Google Earth Imagery 439 samples	Clustering	Width and height	Clustered oil wells	Precision, recall, F1 score, and AP	Faster R-CNN with ClusterRPN = 71%	The proposed method’s running time was higher than the other models, and its accuracy was less than 90%.

**Table 3:** Published research on Fuzzy Logic and Neuro-fuzzy modeling in predictive analytics in the O&G field.

Reference	Models	Temporality	Field	Dataset	Class	Input Parameter	Output Parameter	Performance Metrics	Best Model	Advantages/Disadvantages
[72]	ANFIS, LSSVM-CSA, and Gene Expression Programming	Non-temporal	Oil	The data are confidential.	Prediction	Mixing time (min), MNP dosage (g/L), and oil concentration (ppm)	Oil adsorption capacity (mg/g adsorbent)	R^2^, MPE, and MAPE	LSSVM-CSA R^2^ = 99%	The proposed method was outperformed by the other two models.
[71]	ANFIS and ANFIS+PCA	Non-temporal	Pipeline	Published studies [74,75,76,77,78] 217 samples	Classification	Pipe dimension, burst pressure, pipe wall thickness, defect depth, and defect width	Pressure	RMSE, MAE, and R^2^	ANFIS+PCA R^2^ = 99%	The proposed method outperformed other models and significantly improved the model’s accuracy.
[44]	ANN, SVR, and ANFIS	Non-temporal	Reservoir	CPG’s waterflooding research group at the King Fahd University of Petroleum and Minerals in Saudi Arabia 9000 samples	Clustering	Reservoir heterogeneity degree (V), mobility ratio (M), permeability anisotropy ratio (kz/kx), wettability indicator (WI), production water cut (fw), and oil/water density ratio (DR)	The effectiveness of moveable oil recovery during a flood (RFM)	MAPE, MAE, MSE, and R^2^	ANN	The proposed model had a better accuracy than the other models and had lower a runtime and cost.
[73]	RF, Fuzzy C Means, and Control Chart	Temporal	Well	3W dataset 50,000 samples	Classification	P-PDG, T-PDG, and T-PCK, and grouping of three classes (“normal”, “high fault”, and “high fault”)	Failure detection applications	Total variance	Control chart + RF Specificity = 99% Sensitivity = 100%	The proposed method showed higher sensitivity and specificity.

**Table 4:** Summary of the literature on the application of decision tree, random forest, and hybrid models.

Reference	Models	Temporality	Field	Dataset	Class	Input Parameter	Output Parameter	Performance Metrics	Best Model	Advantages/Disadvantages
[81]	KNN, DT, RF, NB, AdaBoost, XGBoost, and CatBoost	Non-temporal	Pipeline	National Science Foundation (NSF) Critical Resilient Interdependent Infrastructure Systems and Processes (CRISP) 959 samples	Classification	Pipe diameter, wall thickness, defect depth, defect length, yield strength, ultimate tensile strength, and operating pressure	Failure risk pipeline	Precision, recall, and Mean accuracy	XGBoost Accuracy = 85%	The proposed model needs improvement in accuracy.
[82]	LR, RF, SVM, XGBoost, and ANN	Non-temporal	Reservoir	Well log data from North China 1500 samples	Classification	CAL, CNL, AC, GR, PE, RD, RMLL, RS, SP, DEN, DTS, and SP	Shear wave travel time (DTS)	R^2^	XGBoost R^2^ = 99% (training) and 96% (testing)	The best model was significant.
[40]	ELM, SVM, KNN, DT, RF, and EL	Temporal	Transformer	DGA 542 samples	Classification	C_2_H_2_, C_2_H_6_, CH_4_, and H_2_	Power transformer faults	Mean accuracy	EN Accuracy = 78% (Training) and 84% (Testing)	The proposed model’s performance accuracy was not above 90%.
[83]	DT, LDA, GB, Ensemble Tree, LGBM, RF, KNN, NB, LR, QDA, Ridge, and SVM-Linear	Non-temporal	Transformer	DGA 3147 samples	Classification	C_2_H_2_, C_2_H_4_, C_2_H_6_, and CH_4_	Transformer faults	Accuracy, AUC, recall, precision, F1 score, Kappa, MCC, and Processing runtime	QDA Accuracy = 99.29%	The proposed method had the best accuracy classifier model.
[84]	DT	Temporal	Well	KG composition 180 samples	Classification	KG, including hydrogen (H_2_), methane (CH_4_), ethane (C_2_H_6_), ethylene (C_2_H_4_), and acetylene (C_2_H_2_)	Incipient faults in transformer oil.	Accuracy and AUC	DT Accuracy = 62.9%	The current model exhibited potential, and we recommend exploring opportunities for refinement to enhance its overall efficacy.
[85]	LR, DT, RF, KNN, SMOTE, XAI, SHAP, and LIME	Non-temporal	Well	3W 1984 samples	Classification	P-PDG, P-TPT, T-TPT, P-MON- PCK, T-JUS, PCK, P-JUS- CKGL, T-JUS- CKGL, and QGL	Detect anomalies in oil wells	Accuracy, recall, precision, F1 score, and AUC	RF Accuracy = 99.6%, recall = 99.64%, precision = 99.91%, F1 score = 99.77%, and AUC = 1.00%.	The result of the proposed model was significant.
[86]	LDA, QDA, Linear SVC, LR, DT, RF, and Adaboost	Temporal	Well	3W dataset 2000 samples	Classification	P-PDG, P-TPT, T-TPT, P-MON-CKP, and T-JUS-CKP	Undesirable events	F1 score and accuracy	DT Accuracy = 97%	The feature selection did not boost accuracy, and training time was increased with feature selection. The proposed method struggled with class 2 due to limited data and mismatched labels from calculated features.
[110]	DT, ANN, SVM. LR, KNN, and NB	Temporal	Pipeline	External defects of pipelines in the United States 7000 samples	Classification	Consider the defect’s length, breadth, and pipeline’s nominal thickness.	Classification for pipeline corrosion	Accuracy	DT Accuracy = 99.9%	The accuracy of the model was significant to the research.
[89]	LGBM, CatBoost, XGBoost, RF, and NN	Temporal	Crude oil	WTI crude oil 2687 samples	Classification	Gold, silver, crude oil, platinum, copper, the dollar index, the volatility index, and the Euro Bitcoin: Green Energy Resources ESG.	Oil prices	Accuracy and AUC	LGBM and RF	The proposed method indicated superiority over traditional methods.
[90]	GB, RF, and MLR	Non-temporal	Reservoir	Shale gas reservoirs 1400 samples	Prediction	Horizontal wellbore length, hydraulic fracture length, reservoir length, SRV fracture porosity, permeability, spacing, pressure, and total production time.	CO_2_	MSE	RF	The best method surpassed the other method in ML.
[91]	RF, ANN, and FN	Temporal	Drilling	Real time Well-1 data 8983 samples	Classification	Standpipe pressure (SPP), weight on bit (WOB), rotary speed (RS), flow rate (Q), hook load (HL), rate of penetration (ROP), and rotary speed (RS)	Torque and drag (T&D)	R and AAPE	RF	The proposed model had higher accuracy than the other two models.
[92]	RF	Temporal	Reservoir	2D simulation in STARS 240 samples	Prediction	Formation compressibility, volumetric heat capacity, rock, water, oil, and thermal conductivity	Shale barrier	R^2^ and RMSE	RF	The author suggested incorporating more training data and features to improve the proposed method.
[93]	RF, XGBoost, SVM, and LGBM	Non-temporal	Pipeline	Full-scale corroded O&G pipelines 314 samples	Prediction	Depth, length, and width of corrosion defects, wall thickness, pipe diameter, steel grade, and burst pressure	Burst pressure of gas and oil corroded pipelines	R^2^, RMSE, MAE, and MAPE	XGBoost R^2^ = 99% (training) and 98% (testing)	The hybrid proposed model had significantly higher prediction accuracy.
[94]	XGBoost, SVM, and NN	Non-temporal	Pipeline	OLGA data and PIG data 1700 samples	Classification	Geometrical parameters: start of odometry, end of odometry. Latitude, longitude, elevation, and the length of bar. Water volumetric flow rate, continuous velocity, water film shear stress, hold-up, flow regime, pressure, total mass, and volumetric flow rate inclination, temperature, section area, gas mass and volumetric flow rates, gas velocity, wall shear stress, total water mass and flow rate (including vapor),	Internal corrosion in pipeline infrastructures	Mean accuracy and F1 score	XGBoost Accuracy = 62%	The proposed model needs improvement in accuracy.
[95]	RF and CatBoost	Non-temporal	Pipeline	Crude oil dataset 3240 samples	Prediction	Stream composition (NO_2_, NH_2_S, and NCO_2_), pressure (P), velocity (v), and temperature (T)	Corrosion rates	R^2^, MSE, MAE, and RMSE	CatBoost Accuracy = 99.9% (training and testing)	The proposed model’s accuracy outperformed the other models.
[35]	RF and KNN	Temporal	Transformer	DGA 11,400 samples	Classification	Acetylene (CC_2_HH_2_), ethylene (CC_2_HH_4_), ethane (CC_2_HH_6_), methane (CCHH_4_), and hydrogen (HH_2_)	Identify transformer fault types	Mean accuracy	KNN Accuracy = 88%	The proposed model needs an improvement in accuracy.
[96]	XGBoost, CatBoost, LGBM, RF, deep MLN, DBN, and CNN	Non-temporal	Crude oil	Previous studies on CO_2_–oil MMP databank 310 samples	Classification	Crude oil fractions (N_2_, C_1_, H_2_S, CO_2_, and C_2_-C_5_), average critical injection gas temperature (Tcave), reservoir temperature (Tres), and molecular weight of C5+ fraction (MWc5+)	Estimating the MMP of CO_2_–crude oil system	ARD, AARD, RMSE, MPa, and SD	CatBoost R^2^ = 99%	The proposed model confirmed its superiority over other models.
[97]	DF + K-means, RF, SVM, DNN, and DF	Non-temporal	Lithology	Lithology dataset from the Pearl River Mouth Basin 601 samples	Classification	Sandstone (S00), siltstone (S06), grey siltstone (S37), mudstone (N00), sandy mudstone (N01), and limestone (H00).	Lithology identification	Precision, recall, and Fβ	DF + K-means Accuracy = 90%	The baseline method had poor prediction of the minority class, small-amount data label, error labeling, and noisy data.
[20]	GSK- XGBoost	Temporal	Transformer	DGA 128 samples	Classification	Ammonia, acetaldehyde, acetone, ethylene, ethanol, and toluene	Ethanol, ethylene, ammonia, acetaldehyde, acetone, and toluene	Accuracy, precision, recall, F-measure, and beta-factor	GSK- XGBoost Mean accuracy = 50%	The accuracy of the GSK-XGBoost model fell below 90% after employing the developed strategy, while computational time increased.
[98]	LGBM, XGBoost, RF, LR, SVM, NB, KNN, and DT	Non-temporal	Transformer	DGA 796 samples	Classification	H_2_, CH_4_, C2H_2_, C_2_H_4_, and C_2_H_6_	Fault type classification	Accuracy, precision, recall, and F1 score	LGBM Accuracy = 87.06%	The model demonstrated a high level of competence.
[8]	Adaboost, RF, KNN, NB, MLP, and SVM	Non-temporal	Drilling	Drill bit type in Norwegian wells 4312 samples	Classification	Parameter used: Depth as measured, vertical true depth, penetration rate, bit weight, minutes per round, torque, standpipe pressure, mud mass, flow rate, total gas, bit kind, bot quantity, D-exponent, area of total flow, specific mechanical energy, cut depth, and aggressiveness of drill bit.	Drill bit selection	Accuracy, precision, F1 score, recall, MCC, and G-mean	RF Accuracy = 97% (training) and 91% (testing)	The proposed method was more reliable, stable, and accurate than previous models.
[99]	RF	Temporal	Well	3W 1984 samples	Classification	P-PDG, P-TPT, P-PCK, T-PCK, P-JUS-CKGL, T-JUS-CKGL, and gas lift flow	Early fault detection	Accuracy, faulty-normal accuracy (FNACC), real faulty-normal accuracy (RFNACC)	RF Accuracy = 94%	The proposed method had good detection of the early fault.
[87]	One-Directional, CNN, RF, GNN, and QDA	Temporal	Well	3W 1984 samples	Classification	P-PDG, T-TPT, P-MON-CKP, T-JUS-CKP, P-JUS-CKGL, and QGL	Anomalous events in oil	Accuracy, precision, recall, and F1 score	RF Mean accuracy = 95%	The time windows increased.
[88]	RF and PCA	Temporal	Well	3W 1984 samples	Classification	P-PDG, P-TPT, T-TPT, P-MON-CKP, and T-PCK	Anomalous events in oil wells	Accuracy	RF+PCA Accuracy = 90%	The proposed method’s accuracy > 95% for all the classes.
[100]	SVM, LOF, and RF	Temporal	Reservoir	Well log data 37 samples	Clustering	Depth, gamma ray, shallow resistivity, deep resistivity, neutron, density, CALI, and DTS	Sonic (DTC)	R^2^	K-Means+RF R^2^ = 0.92 to R^2^ = 0.98	The proposed hybrid approach outperformed several baseline methods.
[101]	RF	Temporal	Well	Field and well dataset from public dataset U.S. well 934 samples	Clustering	API, On-stream date, Surface latitude and longitude, formation thickness, TVD, lateral length, total proppant mass, total injected fluid volume, API gravity, porosity, permeability, TOC, VClay, oil production rate, gas production rate, water production rate, GPI, and frac fluid	Barrel of oil equivalent (BOE)	RMSE and R^2^	RF RMSE: Train = 7.25% Test = 17.49%	The proposed method needs improvement in accuracy. The RF model was overfitting, and the accuracy of the proposed method must be improved.
[104]	RF with Analog-to-digital converters	Non-temporal	Well	Well logging dataset 100 samples	Clustering	Neutron (CNL), gamma ray (GR), density (DEN), and compressional slowness (DTC)	Well logging data generation	RMSE, MAE, MAPE, and MSE	RF with analog-to-digital converters RMSE = 9%, MAE = 6%, MAPE = 0.031%, and MSE = 86%	The proposed model needs improvement in accuracy for clustering.
[111]	RF	Temporal	Transformer	DPM1 and DPM2 for DGA 2123 samples	Classification	H_2_ (hydrogen), CH_4_ (methane), C_2_H_2_ (acetylene), C_2_H_4_ (ethylene), C_2_H_6_ (ethane), CO (carbon monoxide), CO_2_ (carbon dioxide), O_2_ (oxygen), and N_2_ (nitrogen)	Transformer fault diagnosis	Accuracy	RF Accuracy: DPM1 = 96.2% DPM2 = 96.5%	For the evaluation dataset, the suggested models diagnosed errors with a satisfactory level of performance.
[105]	KNN, Multilayer Perceptron Neural Network, multiclass SVM, and XGBoost	Temporal	Pipeline	Climate change data 81 samples	Classification	Location, time, pipeline age, pipeline material, temperature, humidity, and wind speed.	Gas pipeline	Accuracy, precision, recall, and F1 score	XGBOOST Accuracy = 92%	The model outperformed other models; however, it needs improvement.
[106]	LogitBoost, GBM, XGBoost, AdaBoost, and KNN	Temporal	Well	Lithofacies and well log dataset 399 samples	Classification	GR, CALI, NEU, DT, DEN, RES DEP, RES SLW, PHIT, and SW	Lithofacies predictions	Total Percent Correct (TPC) is an accuracy measure	XGBoost TPC = 97%	The model gave significant results for the proposed method.
[107]	Recursive feature elimination and particle swarm optimization-AdaBoost	Non-temporal	Pipeline	Changshou-Fuling-Wulong-Nanchuan (CN) gas pipeline dataset 3986 samples	Clustering	Landslide susceptibility area, percentage, and historical landslides	Long-distance pipelines	Accuracy, sensitivity, precision, and F1 score	Recursive feature elimination and particle swarm optimization-AdaBoost Accuracy = 90% (training) and 83% (testing)	The proposed model needs improvement in accuracy.
[101]	LSTM, AdaBoost, LR, SVR, DNN, RF, and adaptive RF	Temporal	Crude oil	United states’ Energy Information Administration Brent COP data	Prediction	Shape, location, and scale	Crude oil price (COP)	MAPE, MSE, RMSE, MAE, and EVS	Adaptive RF MAPE = 112.31%; MAE = 52%; MSE = 53%; RMSE =73%; R^2^ = 99%; and EVS = 99%	The proposed model outperformed the others; however, the running time was higher than those of the other models.
[109]	RF and DT	Temporal	Drilling	The data are confidential.	Prediction	WOB, torque, standpipe pressure, drill string rotation speed, rate of penetration, and pump rate	Rock porosity	R^2^, AAPE, and VAF	RF Accuracy = 99% (training) and 90% (testing)	The model stood out for its exceptional performance.
[108]	BayesOpt-XGBoost, and XGBoost	Non-temporal	Reservoir	Equinor Volve Field datasets 2853 samples	Classification	DT, GR, NPHI, RT, and RHOB	Vshale, porosity, horizontal permeability (KLOGH), and water saturation	RMSE and MAE	BayesOpt-XGBoost Accuracy = 93%, precision score = 98%, recall score = 86%, and combined F1 score = 93%	The proposed method was not robust enough to predict all the output.
[103]	RF, KNN, NB, DT, and NN	Temporal	Transformer	New O&G decommissioning dataset from GitHub 1846 samples	Classification	Dimensions, circumference, length, metal, plastic, concrete, residues, environmental expenses, and weight	Predictive decommissioning options	Recall, precision, F1 score, and AUC	RF Accuracy: Full features = 80.06% Redundant removed = 80.66%	The proposed method needs improvement.

**Table 5:** Previous research published on interrelated AI models for predictive analytics in the O&G field.

Reference	Models	Temporality	Field	Dataset	Class	Input Parameter	Output Parameter	Performance Metrics	Best Model	Advantages/Disadvantages
[114]	MLR, SVR, and GPR	Non-temporal	Gas	M6COND and M6GAS 129 samples	Clustering	Condensate–gas ratio, total horizontal lateral length, gas saturation, total organic carbon content, cluster and stage counts, proppant amount, fluid volume, and total horizontal lateral length	Gas well	RMSE and R^2^	GPR	The proposed method needs improvement in accuracy.
[115]	XGBoost, ANN, RNN, MLR, PLR, SVR, DTR, and RFR	Temporal	O&G production	Saudi Aramco of five well reservoirs 1,968 samples	Classification	Location, contact, average permeability, volume, production, pressure ratio between the wellhead and bottom-hole, and production	Oil, gas, and water	R^2^, MAE, MSE, and RMSE	RNN R^2^: Oil = 98% Gas = 87% Water = 92%	The proposed model needs improvement in output.
[116]	MLP, RF, and SVR	Non-temporal	Pipeline	History record of pipeline failure 149,940 samples	Classification	Effects of transportation disruptions on safety and health, the environment and ecology, and equipment maintenance	Natural gas pipeline failure	RMSE, MAE, MSE, and R^2^	RF	The proposed methods had the shortest computing times and best-fitting results.
[117]	SVM	Non-temporal	Reservoir	MMP data 147 samples	Classification	Reservoir temperature, oil composition, and gas composition	Minimum miscibility pressure of CO_2_ and crude oil	MSE	SVM-POLY kernel	The proposed model’s accuracy outperformed the other models.
[22]	RF, ARN, LSTM, Independently Recurrent Neural Network, component-wise gradient	Temporal	Well	3W 1984 samples	Classification	P-PDG, T-TPT, P-TPT, Initial Normal, Steady-state, and transient	Oil well production	Accuracy, precision, recall, F score	ARN Accuracy = 96% Precision = 88% Recall = 84% F-measure = 85%	The proposed model was not robust due to misclassifications for undesirable events for type 3 and type 8.
[118]	SVR-GA-PSO, SVR, SVR-GA, SVR-FA, SVR-PSO, SVR-ABC, SVR-BAT, SVR-COA, SVR-GWO, SVR-HAS, SVR-ICA, and SVR-SFLA	Temporal	Pipeline	Iranian oil fields 340 samples	Classification	Onshore oil and gas pipelines: pit depths, exposure times, pitting start times, operational pressures, temperatures, water cuts, redox potentials, resistivities, pH, concentrations of sulfate and chloride ions, and production rates	Carbon steel corrosion rate	MSE, RMSE, MAE, EVS, R^2^, and RSE	SVR-GA-PSO R^2^ = 99% RMSE = 0.0099 MSE = 9.84 × 10^−5^ MAE = 0.008 RSE = 0.001 EVS = 0.955	The proposed model showed a better result than the other ones.
[119]	BLR, PBBLR, ANN, and Gradient Boosting DT	Non-temporal	Pipeline	SCADA (Supervisory Control and Data Acquisition) system 728 samples	Prediction	Diameter, Reynolds number, transportation distance, and mixed oil length	Actual mixed oil length	RMSE, MAE, and R^2^	PBBLR	The PBBLR method needs improvement on the accuracy of using SCADA dataset to predict actual mixed oil length

**Table 6:** Previous studies on statistical models for predictive analytics modeling in the O&G field.

Reference	Models	Temporality	Field	Dataset	Class	Input Parameter	Output Parameter	Performance Metrics	Best Model	Advantages/Disadvantages
[122]	SARIMA, LSTM, and AR	Temporal	Transformer	DGA 610 samples	Prediction	H_2_, CH_4_, C_2_H_4_, C_2_H_6_, CO, CO_2_, and total hydrocarbon (TH).	Dissolved gas concentration	ARE	SARIMA	The SARIMA method had a good average accuracy
[62]	LSTM and ARIMA	Temporal	Wells	Longmaxi Formation of the Sichuan Basin 3650 samples	Prediction	Date and daily production	Shale gas production	MAE, RMSE, and R^2^	LSTM Accuracy = 0.63%	The accuracy of the model needs improvement.
[123]	GM, FGM, DGGM, ARIMA, PSOGM, and PSO-FDGGM	Temporal	Gas	Quarterly production of natural gas in China	Prediction	Training period and natural gas production	Natural gas production	MAPE	PSO-FDGGM MAPE = 3.19%	The model’s performance was noteworthy and reliable.

**Table 7:** Previous works on the application of ML models for predictive analytics modeling in O&G fields.

Reference	Models	Temporality	Field	Dataset	Class	Input Parameter	Output Parameter	Performance Metrics	Best Model	Advantages/Disadvantages
[124]	Multivariate Empirical Mode Decomposition with Genetic Algorithm, LSSVM-GA, and LSSVM-PSO	Non-temporal	Crude oils	Bubble point pressure and oil formation volume factor 638 samples	Clustering	Temperature (T), oil gravity (API), gas specific gravity (γg), and ratio of gas oil solution	Bubble point pressure and oil formation volume factor of crude oils	RMSE	MELM-PSO	The hybrid proposed model outperformed the empirical method.
[126]	PCA, SVM, and LDA	Temporal	Oil	Real-time oil samples 30 samples	Classification	Pore size remained the same. The capillary flow rate (l2/t) was a function of interfacial properties (γLG and θ) and viscosity (μ).	Oil types	Accuracy	SVM Accuracy = 90%	The proposed model needs improvement in accuracy because the accuracy < 95%.
[127]	MLP-PSO and MLP-GA	Non-temporal	Well log	Three wellbores drilled 22,323 samples	Prediction	Well depth, compressional wave velocity (Vp), shear wave velocity (Vs), bulk density (ρ), and pressure pore (Pp),	Probable depth of casing collapse	R^2^ and RMSE	MLP-PSO	The proposed model outperformed the other models’ accuracy.
[128]	LSSVM-COA, LSSVM-PSO, LSSVM-GA, MLP-COA, MLP-PSO, MLP-GA, LSSVM, and MLP	Non-temporal	Drilling	305 drilled wells in the Marun oil field 2820 samples	Prediction	Northing, easting, depth, meterage, formation type, hole size, WOB, flow rate, MW, MFVIS, retort solid, pore pressure, drilling time, fracture pressure, fan 600/fan 300, gel10min/gel10s, pump pressure, and RPM	Severity of mud loss	R^2^ and RMSE	MLP-GA RMSE = 93%	The accuracy of the proposed model can be improved.
[129]	Hybrid-Physics Guided-Variational Bayesian Spatial-Temporal neural network	Temporal	Gas	Natural gas 600 samples	Prediction	Size of geometry, release point position, release diameter, released gas, volumetric release rate, length of release, and sensor location	Natural gas concentration	R^2^	Hybrid_PG_VBSTnn R^2^ = 99%	The proposed integration enhanced the spatiotemporal forecasting performance.
[125]	CNN, Linear SVM, Gaussian SVM, and SVM+CNN	Temporal	Gas	Leakage dataset 1000 samples	Classification	Methane, ethane, propane, isobutane, butane, helium, nitrogen, hydrogen sulfide, carbon dioxide	Gas pipeline leakage estimation	Accuracy	SVM Accuracy = 95.5%	The model stood out for its exceptional performance.
[130]	LSTM and OCSVM	Temporal	Well	3W 1984 samples	Classification	P-PDG, P-TPT, T-TPT, P-MON-CKP, and T-JUS-CKP	Identify two types of faults	Recall, specificity, and accuracy	OCSVM Accuracy = 91%	The use of feature selection did not improve the classifier accuracy. The proposed model was not robust enough to classify 2 types of wells.
[10]	Ordered Nearest Neighbors, Weighted Nearest Neighbors, LDA, and QDA	Temporal	Well	3W 1984 samples	Classification	P-PDG, P-TPT, T-TPT, P-MON-CKP, T-JUS-CKP, and CLASS	Predicting flow instability	Recall, specificity, and accuracy	ONN Accuracy = 81%	The author suggested investigating another metaheuristic method.
[132]	CNN, SVM, and SVM+CNN	Temporal	Pipeline	Leakage dataset 1000 samples	Prediction	Length, outer diameter, wall thickness, and location in the model	Prediction in tight sandstone reservoirs	Accuracy	SVM+CNN model, achieved 95.5%	The SVM+CNN model outperformed the CNN and SVM
[131]	DT and SVM	Non-temporal	Reservoir	High-resolution FMI data	Classification	Response of logging, pyroclastic lava, normal pyroclastic rock, and sedimentary pyroclastic rock	Lithologic classification of pyroclastic rocks	Accuracy	SVM Accuracy = 98.6%	The SVM accuracy was higher than 95% which is 98.6%
[133]	BAE-OCSVM, CAE-OCSVM, LSTM-AE- OCSVM, RD-OCSVM, RF-OCSVM, PCA-OCSVM, VAE-OCSVM, and LSTM-AE-IF	Temporal	Gas	Data from SCADA 9980 samples	Classification	Diameter, wall thickness, and length	Leakage of natural gas	AUC, accuracy, F1 score, precision, TPR, and FPR	LSTM-AE-OCSVM Accuracy = 98%	The best model achieved higher accuracy, and the author suggested using abnormal data for future work.
[67]	LSTM and GRU	Temporal	Reservoirs	UNISIM-IIH and Volve oilfield 3257 samples	Classification	Oil, gas, water, or pressure	Oil and gas forecasting	SMAPE and R^2^	GRU R^2^ = 99%	The proposed model had the highest accuracy.
[135]	OCSVM, LOF, Elliptical Envelope, and Autoencoder withfeedforward+LSTM	Temporal	Well	3W 1984 samples	Classification	P-PDG, P-TPT, T-TPT, P-MON-CKP, T-JUS-CKP, P-JUS-CKGL, T-JUS-CKGL, QGL, and Label vector	Fault detection	F1 score	LOF F1 score = 85%	The proposed method needs improvement in accuracy.
[134]	K-Means Clustering and KNN	Temporal	Reservoirs	Antrim, Barnett, Eager Ford, Woodford, Fayetteville, Haynesville, and Marcellus 55,623 samples	Clustering	Well location, well depth, well length, and production starting year	EUR predictions	R^2^	K-MC R^2^ = 0.18	The proposed model outperformed the other models using average fitting parameters.
[136]	GS-GMDH	Non-temporal	Well	Oil fields located in the Middle East 2748 samples	Prediction	Laterolog (LLS), photoelectric index (PEF), compressional wave velocity (Vp), porosity (NPHI), gamma ray (spectral) (SGR), density (RHOB), amma ray (corrected) (CGR), shear wave velocity (Vs), caliper (CALI), resistivity (ILD), and sonic transit time (DT)	Pore pressure	RMSE, R^2^, MSE, SI, and ENS	GS-GMDH RMSE = 1.88 psi and R^2^ = 0.9997	GS-GMDH had the best accuracy.
[137]	RF, Gradient Boosting Regressor, Bagging, CNN, KNN, and Deep Hierarchical Decomposition	Temporal	Reservoir	Geological data 180 samples	Classification	Porosity, fracture porosity, fracture permeability, rocky type, net gross, matrix permeability, water relative permeability, formation volume factor, rock compressibility, pressure dependence of water viscosity, gas density, water density, vertical continuity, relative permeability curves, oil–water contact, and fluid viscosity	Oil production, water production, water injection, and liquid production	MAE and SMAPE	Deep Hierarchical Decomposition MAE: OP = 0.76%	The proposed method decreased the computational speed.
[138]	M5P tree model, RF, Random Tree, Reduced Error Pruning Tree, GPR, SVM, and MARS	Non-temporal	Gas	Coriolis flow meter 201 samples	Classification	Wet gas flow rate (kg/h) and absolute gas humidity (g/m^3^)	Estimation of the dry gas flow rate (kg/h)	RMSE, MAE, LMI, and WI	GPR-RBKF MAE = 163.3266 kg/h, RMSE = 483.1359 kg/h, CC = 0.9915 for the dataset used for testing	The best model was superior to the other models, and the author suggested exploring other soft-computing methods.

**Table 8:** Input parameters of undesirable well events from 3W datasets.

Input Parameter of Undesirable Well Events	[86]	[99]	[22]	[73]	[130]	[87]	[88]	[10]	[85]	[135]
P-PDG	√	√	√	√	√	√	√	√	√	√
P-TPT	√		√	√	√	√	√	√	√	√
T-TPT	√		√	√	√	√	√	√	√	√
P-MON-CKP	√			√	√	√	√	√	√	√
T-JUS-CKP	√			√	√	√		√	√	√
T-JUS-CKGL				√					√	√
P-JUS-CKGL				√		√				√
P-CKGL				√						
QGL				√		√			√	√
T-PDG		√								
T-PCK		√					√			

**Table 9:** Input parameters for the fault detection of transformer oil from the DGA dataset.

Input Parameter of Internal Transformer Defects	[35]	[122]	[40]	[83]	[20]	[98]	[59]	[139]	[65]	[111]
Acetylene (C_2_H_2_)	√		√	√		√	√	√	√	√
Ethylene (C_2_H_4_)	√	√		√	√	√	√	√	√	√
Ethane (C_2_H_6_)	√	√	√	√		√	√	√	√	√
Methane (CH_4_)	√	√	√	√		√	√	√	√	√
Hydrogen (H_2_)	√	√	√			√	√	√	√	√
Total Hydrocarbon (TH)		√								
Carbon Monoxide (CO)		√					√	√	√	√
Carbon Dioxide (CO_2_)		√					√	√	√	√
Ammonia (NH_3_)					√					
Acetaldehyde (CH_3_CHO)					√					
Acetone (CH_32_CO)					√					
Nitrogen (N_2_)										√
Ethanol (CH_3_CH_2_OH)					√					

**Table 10:** Input parameters of well logging.

Input Parameter of Well Logging	[64]	[106]	[104]	[140]	[100]	[108]
Gamma Ray (GR)	√	√	√	√	√	√
Sonic (Vp)	√			√		
Deep and Shallow Resistivities (LLD and LLS)	√			√		
Neuro-porosity (NPHI)	√					√
Density (RHOB)	√			√	√	√
Caliper (CALI)		√		√	√	
Neutron (NEU)		√	√		√	
Sonic Transit Time (DT)		√		√	√	√
Bulk Density (DEN)		√	√			
Deep Resistivity (RD)					√	
True Resistivity (RT)						√
Shallow Resistivity (RES SLW)		√			√	
Total Porosity (PHIT)		√				
Water Saturation (SW)		√				
Compressional Slowness (DTC)			√			
Depth					√	

**Table 11:** A summary of each ML method’s accuracy for predictive analytics in the O&G industry from previous studies.

ML Methods	Model Variants	Model Performance (%)
Artificial Neural Network	LWQPSO-ANN	95
	ANN	93
	ANN	99.6
	ANN	90
	DNN	146
	ANN+PSO	99
	ANN	97
	MTGNN	92
	Multilayer Perceptron Backpropagation	89
	GA backpropagation neural network	97
	MLP	10
	DE+ELM	49.7
Deep Learning	DCNN+LSTM	99.37
	LSTM	94
	KNN+SMOTE	98
	DL	99
	GRU	99
	Faster R-CNN+ClutserRPN	71
Fuzzy Logic and Neuro-fuzzy	LSSVM+CSA	99
	ANFIS+PCA	99
	Control Chart+RF	99
Decision Tree, Random Forest, and Hybrid	XGBOOST	85
	XGBOOST	96
	EL	84
	QDA	99.29
	DT	62.9
	RF	99.6
	DT	97
	DT	99.9
	XGBOOST	62
	CATBOOST	99.9
	KNN	88
	CATBOST	99
	DF+K-MEANS	90
	GSK+XGBOOST	50
	LGBM	87.06
	RF	91
	RF	94
	RF	95
	RF+PCA	90
	K-MEANS+RF	98
	RF	17.49
	RF+Analog-to-digital converters	9
	RF	96
	XGBOOST	92
	XGBOOST	97
	Recursive feature elimination+PSO+ADABOOST	83
	Adaptove+RF	73
	RF	90
	BayesOpt+XGBOOST	93
	RF	80.06
Interrelated AI	RNN	98
	ARN	96
	SVR+GA+PSO	99
Statistical model	ARIMA	63
ML model utilized for predictive analytics in the O&G field	SVM	90
	MLP+GA	93
	Hybrid-Physics Guided-Variational Bayesian Spatial-Temporal Neural Network	99
	SVM	95.5
	OCSVM	91
	ONN	81
	SVMCNN	95.5
	AVM	98.6
	LSTM+AE+OCSVM	98
	GRU	99
	LOF	85
	K+MC	18
	Deep Hierarchical Decomposition	76

## Data Availability

This study did not report any data.

## Abbreviations

Abbreviation	Definition	Abbreviation	Definition
RF	Random Forest	DNN	Deep Neural Network
GAM	Generalized Additive Model	MELM	Multivariate Empirical Mode Decomposition
NN	Neural Network	ANFIS	Adaptive Neuro-Fuzzy Inference System
SVR-GA	Support Vector Regression with Genetic Algorithm	SOM	Self-Organizing Map
SVR-PSO	Support Vector Regression with Particle Swarm Optimization	ANN	Artificial Neural Network
SVR-FFA	Support Vector Regression with Firefly Algorithm	MRGC	Maximum Relevant Gain Clustering
GB	Gradient Boosting	CatBoost	Categorical Boosting
LSSVM-CSA	Least Squares Support Vector Machine with Cuckoo Search Algorithm	MLR	Multiple Linear Regression
AHC	Agglomerative Hierarchical Clustering	SVM	Support Vector Machine
XGBoost	Extreme Gradient Boosting	FN	Fuzzy Network
GPR	Gaussian Process Regression	LDA	Linear Discriminant Analysis
LWQPSO-ANN	Linearly Weighted Quantum Particle Swarm Optimization with Artificial Neural Network	LSSVM	Least Squares Support Vector Machine
PCA	Principal Component Analysis	DL	Deep Learning
MLP-ANN	Multilayer Perceptron with Artificial Neural Network	MLSTM	Multilayer Long Short-Term Memory
MLP-PSO	Multilayer Perceptron with Particle Swarm Optimization	GRU	Gated Recurrent Unit
DT	Decision Tree	AdaBoost	Adaptive Boosting
LSTM	Long Short-Term Memory	LSTM-AE-IF	Long Short-Term Memory Autoencoder with Isolation Forest
KNN	k-Nearest Neighbors	DNN	Deep Neural Network
NB	Naive Bayes	CNN	Convolutional Neural Network
GP	Genetic Programming	O&G	Oil and Gas
ELM	Extreme Learning Machine	AI	Artificial Intelligence
DF	Deep Forest	MSE	Mean Squared Error
QDA	Quadratic Discriminant Analysis	MAPE	Mean Absolute Percentage Error
ML	Machine Learning	AAPE	Arithmetic Average Percentage Error
DGA	Dissolved Gas Analysis	SMAPE	Symmetric Mean Absolute Percentage Error
RMSE	Root Mean Squared Error	RSE	Relative Squared Error
MAE	Mean Absolute Error	RFR	Random Forest Regression
AUC	Area Under the Curve	FNACC	Faulty-Normal Accuracy
ARE	Absolute Relative Error	TPC	Total Percent Correct
EVS	Explained Variance Score	VAF	Variance Accounted For
DTR	Decision Tree Regression	WI	Weighted Index
PLR	Polynomial Linear Regression	LMI	Linear Mean Index
SNR	Signal-to-Noise Ratio	AP	Average Precision
RFNACC	Real Faulty-Normal Accuracy	MAP	Mean Average Percentage
RMSPE	Root Mean Square Percentage Error	ARD	Absolute Relative Difference
MARE	Mean Absolute Relative Error	Mpa	Megapascal
SI	Severity Index	P-JUS-CKGL	Pressure Downstream of Gas Lift Choke
ENS	Energy Normalized Score	P-CKGL	Pressure Downstream of Gas Lift Choke (CKGL)
MPE	Mean Percentage Error	QGL	Gas Lift Flow Rate
R	Correlation of Coefficient	T-PDG	Temperature at the Permanent Downhole Gauge Sensor
AARD	Average Absolute Relative Deviation	T-PCK	Temperature Downstream of the Production Choke
P-PDG	Pressure at Permanent Downhole Gauge (PDG)	LSB	Least Square Boosting
P-TPT	Pressure at Temperature/Pressure Transducer (TPT)	PLS	Partial Least Squares
T-TPT	Temperature at TPT	FPM	Feature Projection Model
P-MON-CKP	Pressure Upstream of Production Choke (CKP)	FP-DNN	Feature Projection-Deep Neural Network
T-JUS-CKP	Pressure Downstream of CKP	GNN	Graph Neural Network
T-JUS-CKGL	Temperature Downstream of CKGL	MLP	Multilayer Perceptron
FP-PLS	Feature Projection-PLS	Bi-LSTM	Bidirectional Long Short-Term
MGGP	Multi-Gene Genetic Programming	SHAP	Shapley Additive Explanation
xNES	Exponential Natural Evolution Strategies	LR	Logistic Regression
RNN	Recurrent Neural Network	LOF	Local Outlier Factor
LGBM	Light Gradient Boosting Machine	ICA	Imperialist Competitive Algorithm
SMOTE	Synthetic Minority Oversampling Technique	SFLA	Shuffled Frog-Leaping Algorithm
LIME	Local Interpretable Model-Agnostic Explanations	SA	Simulated Annealing
XAI	Explainable Artificial Intelligence	PBBLR	Physics-Based Bayesian Linear Regression
GSK	Gaining-Sharing Knowledge-Based Algorithm	ARIMA	Autoregressive Integrated Moving Average
BayesOpt-XGBoost	Bayesian oOptimization XGBoost	GM	Generalized Method of Moments
FA	Firefly Algorithm	PSO-FDGGM	PSO-Based Data Grouping Grey Model with a Fractional Order ccumulation
COA	Cuckoo Optimization Algorithm	PSOGM	PSO for Grey Model
GWO	Grey Wolf Optimizer	LSSVM	Least Square Support Vector Machine
HAS	Harmony Search	GA	Genetic Algorithm
BLR	Bayesian Linear Regression	OCSVM	One-Class Support Vector Machine
SARIMA	Seasonal Autoregressive Integrated Moving Average	BAE	Basic Autoencoder
GM	Grey Model	CAE	Convolutional Autoencoder
FGM	Fractional Grey Model	AE	Autoencoder
DGGM	Data Grouping-Based Grey Modeling Method	VAE	Variational Autoencoder
GPR	Gaussian Process Regression	MARS	Multivariate Adaptive Regression Spline
